# Valorization of the Organic Fraction of Municipal Solid Waste (OFMSW): Biochar Production, Activation, and Characterization for Hexavalent Chromium Adsorption

**DOI:** 10.3390/molecules31213912

**Published:** 2026-10-29

**Authors:** Daniel A. Rivadeneira, Nilo M. Robles Carrillo, Brigitte Rivilla Cueva, Cristhian Velalcázar-Rhea, Michelle Romero

**Affiliations:** 1 Instituto de Investigación Geológico y Energético (IIGE), Quito 170518, Ecuador; daniel.rivadeneira@geoenergia.gob.ec (D.A.R.); brigitte.rivilla@geoenergia.gob.ec (B.R.C.); cristhian.velalcazar@geoenergia.gob.ec (C.V.-R.); michelle.romero@geoenergia.gob.ec (M.R.); 2 Alimentos, Compuestos Orgánicos, Materiales, Microbiología Aplicada y Energía (ACMME), Facultad de Ingeniería Química, Universidad Central del Ecuador, Quito 170129, Ecuador

**Keywords:** lignocellulosic waste, organic fraction of municipal solid waste, pyrolysis kinetics, activated biochar, hexavalent chromium Cr(VI), adsorption kinetics

## Abstract

The valorization of the organic fraction of municipal solid waste (OFMSW), an underexplored lignocellulosic waste, represents a sustainable strategy for the production of adsorbents aimed at wastewater remediation, particularly against hexavalent chromium Cr(VI) from tannery effluents, a persistent environmental hazard in regions such as Tungurahua, Ecuador, where concentrations widely exceed regulatory limits. This study investigates the valorization of OFMSW pyrolyzed at 575 °C and subsequently activated by physical treatment with CO_2_ to produce an activated biochar (BCA-OFMSW) for remediating aqueous solutions containing Cr(VI). The structural, ultimate, and textural properties were characterized before and after activation, and batch adsorption experiments evaluated the effects of pH, adsorbent dosage, initial concentration, and contact time, followed by equilibrium and kinetic modeling. Activation increased the surface area by more than two orders of magnitude (NLDFT = 1159.58 m^2^ g^−1^). Maximum removal occurred at pH 2, consistent with the alkaline point of zero charge of the material (pH_PZC_ = 11.47). The Freundlich model (R^2^ = 0.9590) fitted the equilibrium data better than the Langmuir model (R^2^ = 0.8453; qm = 58.86 mg g^−1^), and the Elovich model gave the highest R^2^ among the kinetic models tested (0.9654). Adsorption increased with temperature (ΔH° = +9.08 kJ mol^−1^, an approximate estimate derived from the Freundlich constant). The pH dependence suggests a contribution of electrostatic attraction; post-adsorption XRD and EDS show chromium at the surface but do not identify its oxidation state, so Cr(VI) reduction could not be assessed. These results indicate that OFMSW-derived activated biochar is a promising adsorbent for Cr(VI)-contaminated effluents.

## 1. Introduction

Global economic development and ongoing population growth in urban areas have resulted in solid waste generation. According to the United Nations (UN), global municipal solid waste (MSW) production may reach approximately 3.8 billion tons by 2050 [1]. Inefficient management of this waste, primarily from productive activities and human consumption, presents a significant environmental challenge, leading to greenhouse gas emissions and the contamination of soil and water resources [2,3]. Due to its high availability and chemical composition, OFMSW constitutes a widely available, low-cost precursor for thermochemical conversion into value-added carbonaceous materials. In this context, comprehensive valorization of such waste is an essential strategy for advancing toward a circular and decarbonized economy, in accordance with the Sustainable Development Goals (SDGs).

The OFMSW is a complex, heterogeneous matrix rich in biopolymers and organic compounds. Within this fraction, lignocellulosic biomass constitutes a significant proportion. It is structurally composed of cellulose, hemicellulose, and lignin, along with minor components such as proteins, pectins, and inorganic matter. Given its high availability and energy density, the OFMSW represents a low-cost, high-value precursor for thermochemical conversion [4,5].

A sustainable application of lignocellulosic waste, such as OFMSW, involves using it in conversion processes aimed at producing biofuels. Among the available conversion technologies, gasification, liquefaction, direct combustion, and pyrolysis are particularly noteworthy [6]. Pyrolysis is recognized as a highly efficient method for biomass valorization. This process involves the thermal decomposition of organic matter in an inert atmosphere at temperatures ranging from 200 °C to 900 °C [7], resulting in products such as biochar, bio-oil, and non-condensable gases [8]. Operational parameters such as temperature, heating rate, and residence time, as well as the biomass’s macromolecular composition, influenced the distribution and physicochemical characteristics of these products. For example, the thermal degradation of cellulose and hemicellulose favors the formation of bio-oil and biogas, whereas the cross-linking of aromatic rings in lignin promotes the formation of biochar with a high degree of aromatization and stability [9].

It is imperative to highlight that biochar is a porous, alkaline, carbonaceous material distinguished by a recalcitrant structure, a high specific surface area, and the presence of various oxygenated functional groups (such as hydroxyls, carboxyls, and carbonyls) on its surface [10]. These attributes confer upon it an exceptional adsorption capacity, thereby rendering it a promising material for environmental remediation, carbon dioxide capture, and contaminant removal in aqueous systems [11].

The application of biochar is particularly relevant in countries like Ecuador, where heavy metal contamination of surface waters, soils, and food is a severe issue resulting from industrial and agricultural activities [12]. Among aquatic pollutants, hexavalent chromium Cr(VI) represents an urgent environmental and public health concern globally and regionally. Derived from industrial operations such as electroplating, steel manufacturing, dye synthesis, petroleum refining, and leather tanning, Cr(VI) is distinguished by its high aqueous solubility, mobility, and acute toxicity, mutagenicity, and carcinogenicity in living organisms [13,14].

In Ecuador, this issue is particularly critical in the province of Tungurahua, where intense tannery activities discharge industrial effluents into river basins with Cr(VI) levels ranging from 52.3 to 393.9 mg L^−1^ [14], drastically exceeding the maximum permissible limit of 0.5 mg L^−1^ established by current national environmental regulations [15].

Conventional methods for removing heavy metals from water include chemical precipitation, ion exchange, membrane filtration, and electrochemical processes; however, these methods have significant limitations, including high operational costs, intensive energy consumption, and the generation of toxic secondary sludge [16]. As an alternative, adsorption has emerged as a highly efficient, cost-effective, and sustainable technology [17]. The removal mechanism relies on retaining metal ions in the liquid phase onto the surface of a solid material (adsorbent) through physical or chemical interactions [18]. Process efficiency depends on multiple factors, including adsorbent type, solution pH, particle size, contact time, initial metal concentration, and the presence of competing ions [19].

To enhance heavy metal binding performance, recent research has explored various functionalized or modified biochars, including iron nanocomposites, nitrogen-doped matrices, and magnetically modified agricultural residues [20,21,22]. For instance, Zn/Fe-modified jujube branch biochar achieved a maximum Cr(VI) adsorption capacity of 27.85 mg g^−1^ [20], while nitrogen-doped wheat straw biochar loaded with zero-valent iron nanoparticles reached capacities up to 100.9 mg g^−1^ under controlled laboratory conditions [22]. Furthermore, iron-modified rice straw biochar demonstrated high efficiency via coupled electrostatic attraction and reduction pathways [21].

In addition, several studies have reported the use of biomass-derived biochars for this purpose Masuku et al. [23] evaluated a biochar from pinecone waste for Cr(VI) removal in 50 mL of an aqueous solution adjusted to pH 2 with an initial concentration of 100 mg L^−1^. Under these conditions, 0.25 mg of adsorbent and a 120 min contact time achieved 69% Cr(VI) removal, demonstrating the material’s efficacy in highly acidic environments. Similarly, Barczak and Januszewicz [24] studied activated biochars derived from Acorus calamus for Cr(VI) removal, using solutions with initial concentrations ranging from 10 to 150 mg L^−1^. The authors evaluated the effects of pH, activated carbon dosage, and contact time, observing 63% removal within the first 2 min, which increased to 88% at 120 min under optimal operating conditions: pH 2, an initial Cr(VI) concentration of 50 mg L^−1^, and an adsorbent dose of 0.35 g L^−1^. Also, Abewaa et al. [25] investigated activated biochar synthesized from Rumex abyssinicus, which has a high specific surface area (1722.9 m^2^ g^−1^). In their adsorption tests, they achieved a Cr(VI) removal efficiency of 95.97% using 0.6 mg of adsorbent in 100 mL of a solution with an initial concentration of 70 mg L^−1^ at pH 2 and a contact time of 60 min.

The novelty of this study lies in the direct valorization of a raw, unhomogenized municipal market residue (OFMSW collected from wholesale market waste) into an activated biochar (BCA-OFMSW) through a two-step thermochemical process: pyrolysis at 575 °C followed by physical CO2 activation at 820 °C. Unlike more uniform agricultural biomasses, OFMSW is a heterogeneous protein-lignocellulosic matrix, and its performance as a Cr(VI) adsorbent is evaluated here.

This study aims to synthesize and characterize an activated biochar from OFMSW and to evaluate its potential for Cr(VI) removal from aqueous media. The feedstock was characterized by physicochemical analysis and thermogravimetry, and the biochar (BC-OFMSW) and activated biochar (BCA-OFMSW) by FTIR-ATR, XRD, SEM, and N2 physisorption (BET and NLDFT). The effects of pH, contact time, adsorbent dosage, and initial Cr(VI) concentration were then studied in batch assays, followed by equilibrium, kinetic, and temperature-dependence analyses. Finally, the post-adsorption material (BCA-Cr) was examined by XRD and SEM-EDS.

## 2. Results and Discussion

The data supporting the findings of this study can be found in the Supplementary Materials.

### 2.1. Physicochemical Characterization of OFMSW

The physicochemical characteristics of the OFMSW feedstock are summarized in Table 1. The material presented a moisture content of 3.41 ± 0.05% (w.b.), an ash content of 16.90 ± 0.07% (d.b.), and a volatile matter content of 68.28 ± 0.12% (d.b.). Fixed carbon, calculated by difference, represented 11.41% (d.b.). The higher heating value (HHV) was 15.70 ± 0.08 MJ kg^−1^.

The ultimate analysis performed on a moisture- and ash-free basis showed carbon, hydrogen, nitrogen, sulfur, and oxygen contents of 32.59 ± 0.95%, 5.34 ± 0.46%, 10.76 ± 0.65%, 0.06 ± 0.09%, and 51.24 ± 1.97%, respectively. The calculated H/C and O/C atomic ratios were 1.95 and 1.18.

The compositional analysis revealed cellulose, hemicellulose, and lignin contents of 26.35 ± 0.74%, 10.46 ± 0.12%, and 21.47 ± 0.41%, respectively. Water extractives accounted for 37.04 ± 0.67%, whereas ethanol extractives represented 4.68 ± 0.11% of the dry biomass (Table 1).

### 2.2. Thermal Behavior of OFMSW

**Figure 1:**
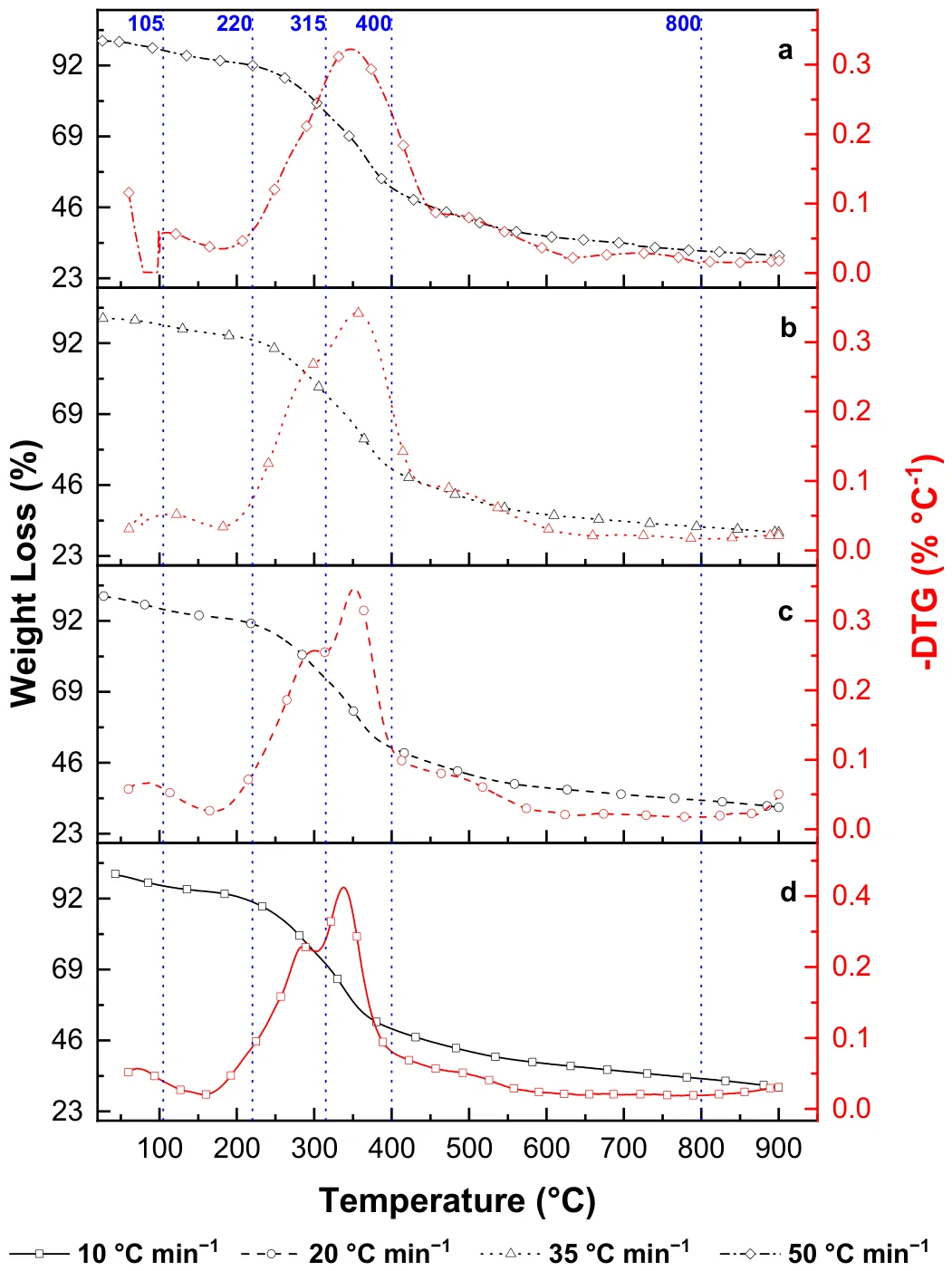
Thermogravimetric (TGA) and derivative thermogravimetric (DTG) curves of OFMSW under pyrolytic conditions; (**a**) heating rate 50 °C min^−1^; (**b**) heating rate 35 °C min^−1^; (**c**) heating rate 20 °C min^−1^; (**d**) heating rate 10 °C min^−1^.

The TGA curves showed mass loss in several stages: a small loss at low temperature, a larger one as the organic fraction decomposed, and a slower loss at higher temperatures that leveled off at a residual mass. The DTG curves showed overlapping peaks and shoulders at every heating rate, with the fastest mass loss in the main devolatilization region. OFMSW therefore decomposes in several overlapping stages (Figure 1).

Below 105 °C, the mass loss corresponds to moisture. Between 105 and 220 °C, it is attributed to water- and ethanol-soluble extractives (sugars, organic acids, and pectins), which weigh far more in a mixed waste such as OFMSW than in woody feedstocks. Hemicellulose decomposes between 220 and 315 °C and cellulose mainly between 315 and 400 °C, in line with the DTG peaks reported for orange peel (210, 319, and 466 °C) and peanut shell (280, 340, and 416 °C) [26]. Above 400 °C, lignin breakdown, aromatization, and char formation drive the mass loss. Single-source biomass shows discrete DTG peaks, whereas OFMSW shows overlapping ones, which reflects its heterogeneous composition, as also reported for mixed precursors such as municipal sewage sludge [27,28].

### 2.3. Thermogravimetric Kinetics of Pyrolysis

The kinetic analysis of OFMSW pyrolysis was performed over the conversion range of 0.20–0.70 using the KAS and FWO isoconversional methods. Within this interval, both models provided the most consistent linear regressions.

**Figure 2:**
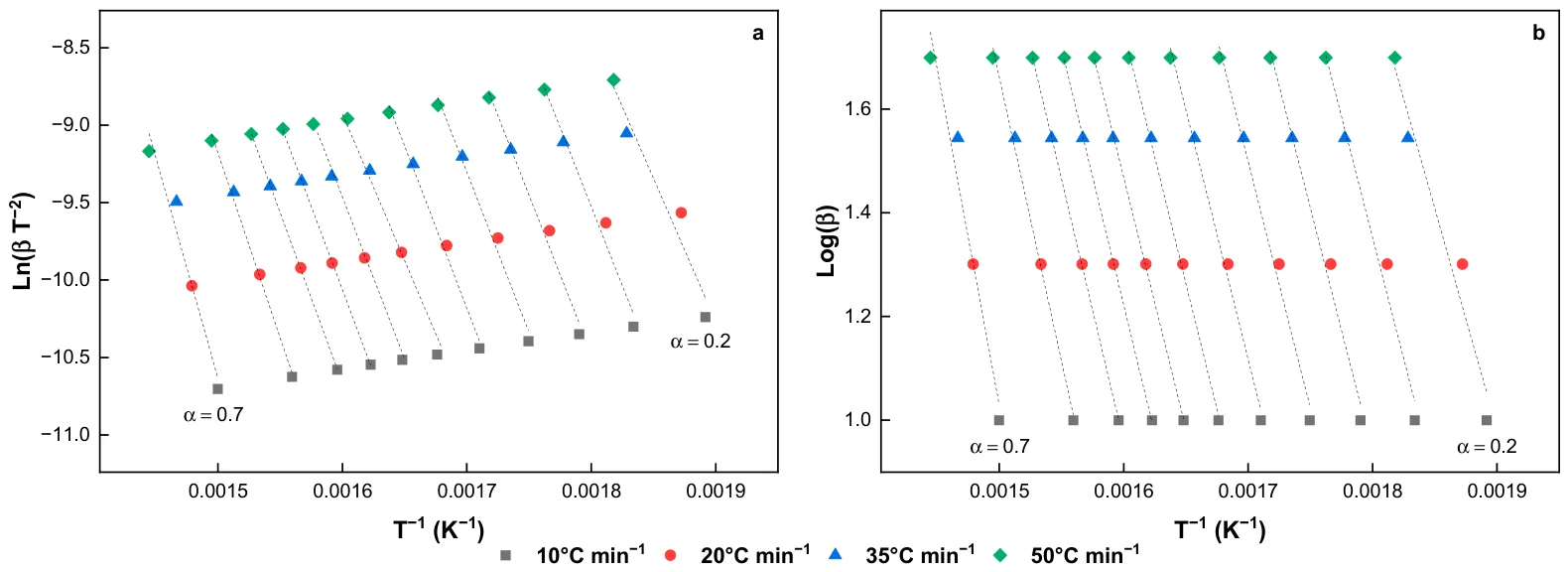
Apparent activation energy (Eₐ) as a function of conversion (α) for OFMSW pyrolysis over the conversion range 0.20–0.70. (**a**) KAS model; (**b**) FWO model.

The apparent activation energy varied with conversion for both methods (Figure 2). For the KAS model, activation energy increased from 153.58 kJ mol^−1^ at α = 0.20 to 237.36 kJ mol^−1^ at α = 0.70.

A similar trend was observed for the FWO model. Activation energy increased from 154.57 kJ mol^−1^ at α = 0.20 to 236.46 kJ mol^−1^ at α = 0.70. Across most conversion levels, the activation energies obtained by the FWO method were slightly higher than those calculated using the KAS approach.

The average activation energies were 179.05 ± 22.76 kJ mol^−1^ for KAS and 181.63 ± 21.13 kJ mol^−1^ for FWO. Figure 2 presents the variation in activation energy with conversion. The progressive increase in Ea with α indicates that pyrolysis proceeds through several parallel and consecutive reactions rather than a single-step process, consistent with the heterogeneous composition of OFMSW (Table 1).

### 2.4. Thermodynamic Parameters of Pyrolysis

Table 2 presents the thermodynamic parameters calculated using the KAS and FWO isoconversional methods.

For the KAS model, the average activation energy was 179.05 ± 22.76 kJ mol^−1^, while the mean value of lnA was 35.70 ± 2.77 s^−1^. The corresponding average values of ΔH, ΔG, and ΔS were 173.99 ± 22.47 kJ mol^−1^, 207.28 ± 37.39 kJ mol^−1^, and −54.27 ± 22.83 J mol^−1^ K^−1^, respectively.

For the FWO model, the average activation energy was 181.63 ± 21.13 kJ mol^−1^, and the mean lnA value was 35.68 ± 2.45 s^−1^. The average values of ΔH, ΔG, and ΔS were 176.57 ± 20.83 kJ mol^−1^, 209.93 ± 35.13 kJ mol^−1^, and −54.10 ± 20.02 J mol^−1^ K^−1^, respectively.

The coefficients of determination remained high throughout the conversion interval evaluated. Average R^2^ values were 0.9856 for the KAS model and 0.9878 for the FWO model (Table 2). The positive ΔG values reflect the energy barrier associated with the formation of the activated complex, and the negative ΔS values suggest that the activated complex is more ordered than the reactants.

### 2.5. Physicochemical Composition of OFMSW and BC-OFMSW and Yield of BC-OFMSW and BCA-OFMSW

The final composition of OFMSW and BC-OFMSW is presented in Table 3. After pyrolysis, the carbon content increased from 32.59 ± 0.95% to 41.75 ± 5.27% (d.b.). The hydrogen content increased from 5.34 ± 0.46% to 8.97 ± 2.57% (d.b.), while nitrogen decreased from 10.76 ± 0.65% to 1.19 ± 0.08% (d.b.). No sulfur was detected in BC-OFMSW. The oxygen content decreased from 51.24 ± 1.97% to 48.10 ± 7.92% (d.b.).

The atomic H/C ratio increased from 1.95 in OFMSW to 2.56 in BC-OFMSW, while the O/C ratio decreased from 1.18 to 0.86 (Table 3). The ash content was 16.90 ± 0.07% for OFMSW and 37.43 ± 0.53% for BC-OFMSW. The increase in the H/C ratio after pyrolysis (from 1.95 to 2.56) runs against the decrease normally associated with carbonization and aromatic ring condensation [28]. The carbon content of BC-OFMSW (41.75 ± 5.27%) also has a large standard deviation, and chars with high ash content tend to show low carbon values [27,29]. These elemental values, obtained from duplicate measurements on a char with 37.43% ash, should therefore be interpreted with caution. In contrast, the decrease in nitrogen (from 10.76 to 1.19%) and in the O/C ratio (from 1.18 to 0.86) is consistent with the volatilization of nitrogenous compounds such as proteins, which decompose at 200–500 °C in organic waste [29], and of oxygenated groups during pyrolysis at 575 °C.

The pyrolysis yield of BC-OFMSW was 33.39 ± 1.20%, while the activation yield of BCA-OFMSW was 30.75 ± 1.88% (Table 4).

### 2.6. Surface Chemistry: FTIR-ATR of OFMSW, BC-OFMSW and BCA-OFMSW

**Figure 3:**
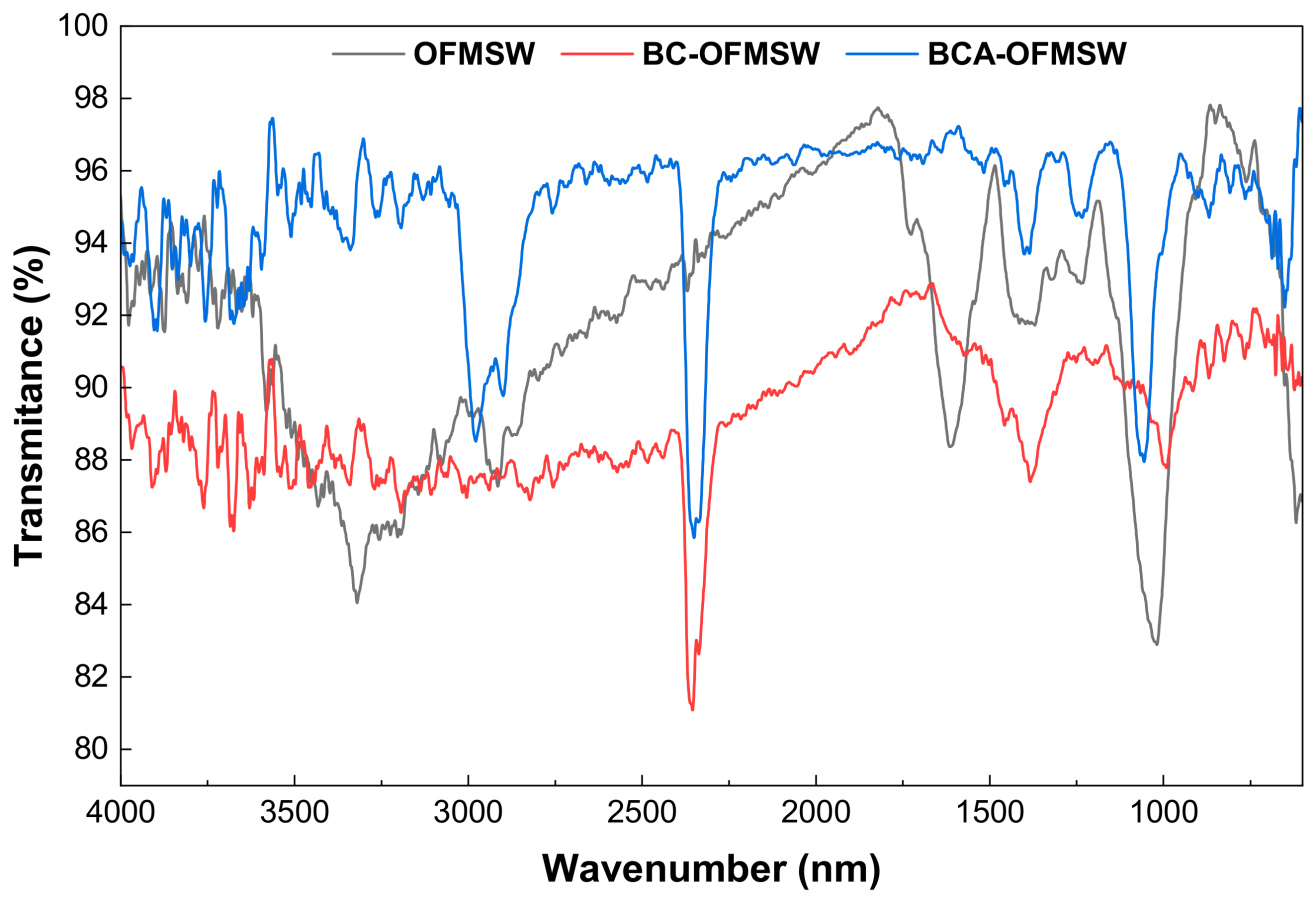
FTIR spectra of OFMSW and the corresponding carbon materials.

The FTIR—ATR spectra of OFMSW, BC-OFMSW, and BCA-OFMSW are shown in Figure 3. The raw feedstock displays the typical bands of lignocellulosic biomass: a broad O–H stretching band at about 3300–3400 cm^−1^ (moisture and biopolymer hydroxyls), a band near 1600–1650 cm^−1^ from C=O and aromatic C=C, and a strong absorption at 1000–1100 cm^−1^ from C–O–C and Si–O–Si bonds [29,30]. After pyrolysis at 575 °C (BC-OFMSW), most of these bands weaken markedly. The broad O–H band practically disappears and the 1600–1650 cm^−1^ band becomes very weak, which indicates that hydroxyl and other labile oxygenated groups were lost through dehydration and deoxygenation. The 1000–1100 cm^−1^ absorption stays weak in BC-OFMSW but becomes strong again after CO_2_ activation (BCA-OFMSW). This is probably because ash-derived inorganic components, mainly silicates, become concentrated in the carbon matrix as organic mass is lost. Overall, pyrolysis and activation progressively removed the labile oxygenated groups of the feedstock, while the Si–O and C–O framework remained.

### 2.7. Structural Characterization (XRD) of OFMSW, BC–OFMSW and BCA–OFMSW

**Figure 4:**
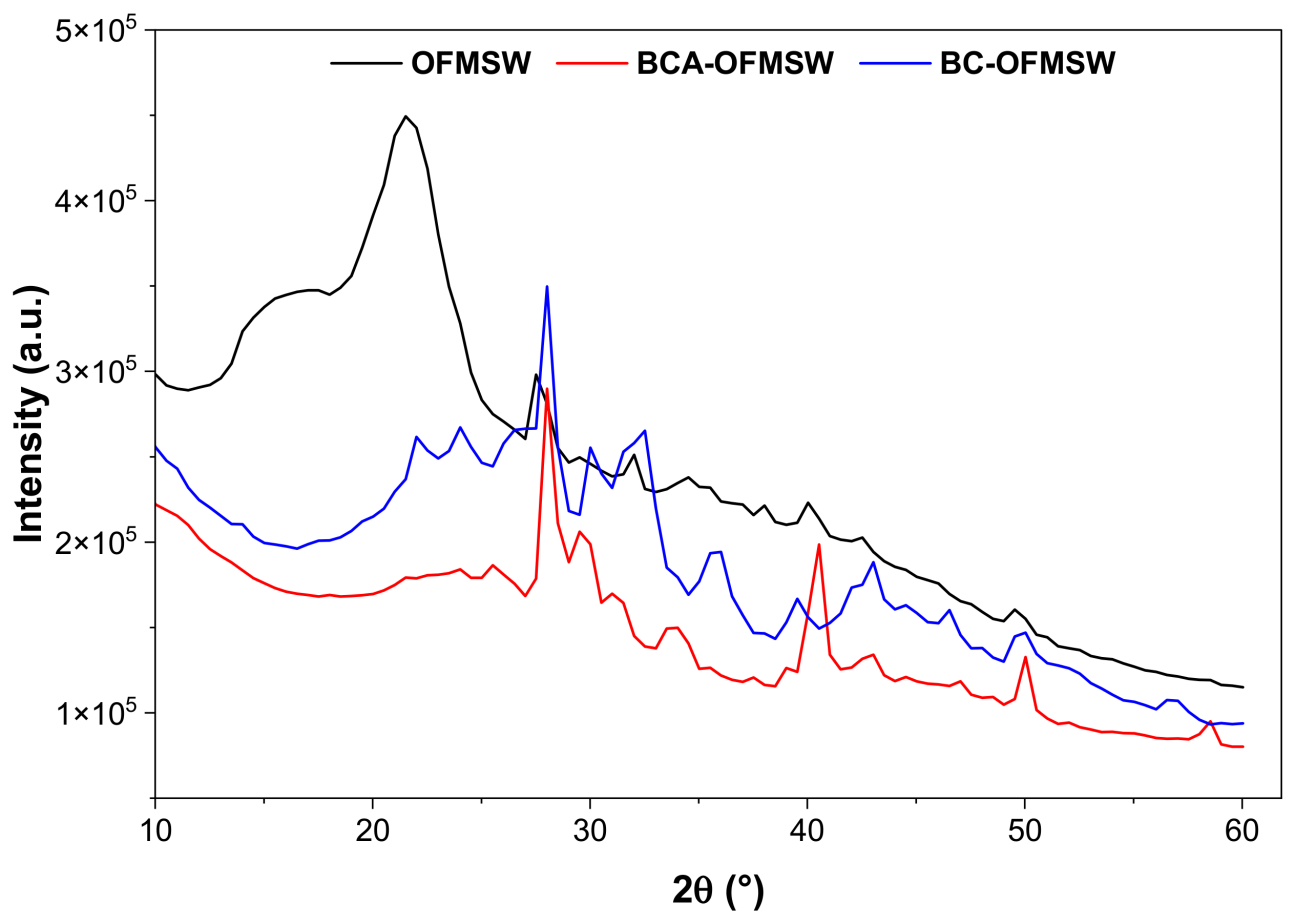
X-ray diffraction (XRD) patterns of OFMSW and the derived carbon materials.

The XRD patterns of OFMSW, BC-OFMSW, and BCA-OFMSW are shown in Figure 4. The raw feedstock exhibits a broad amorphous halo centered near 2θ ≈ 21–22°, corresponding to the disordered biopolymer matrix (pectins, hemicellulose, and extractives) overlapping the cellulose structure in unprocessed market waste [29,30]. After pyrolysis at 575 °C, BC-OFMSW shows sharp reflections at 2θ ≈ 28°, 40°, and 50°, which are consistent with sylvite (KCl), a phase also reported in the XRD patterns of plant-derived biochars [29,30]. This assignment is based on peak positions only, and the 50° reflection lies at the upper limit of the scanned range (0–50°). The thermal loss of volatile organic matter concentrates the inorganic fraction, as shown by the higher ash content of BCA-OFMSW (45.26 ± 0.13%) compared with BC-OFMSW (37.43 ± 0.53%). In BCA-OFMSW, the 28° reflection remains, while those at 40° and 50° become weaker and broader.

### 2.8. Morphological Characterization (SEM) of OFMSW and BC-OFMSW

**Figure 5:**
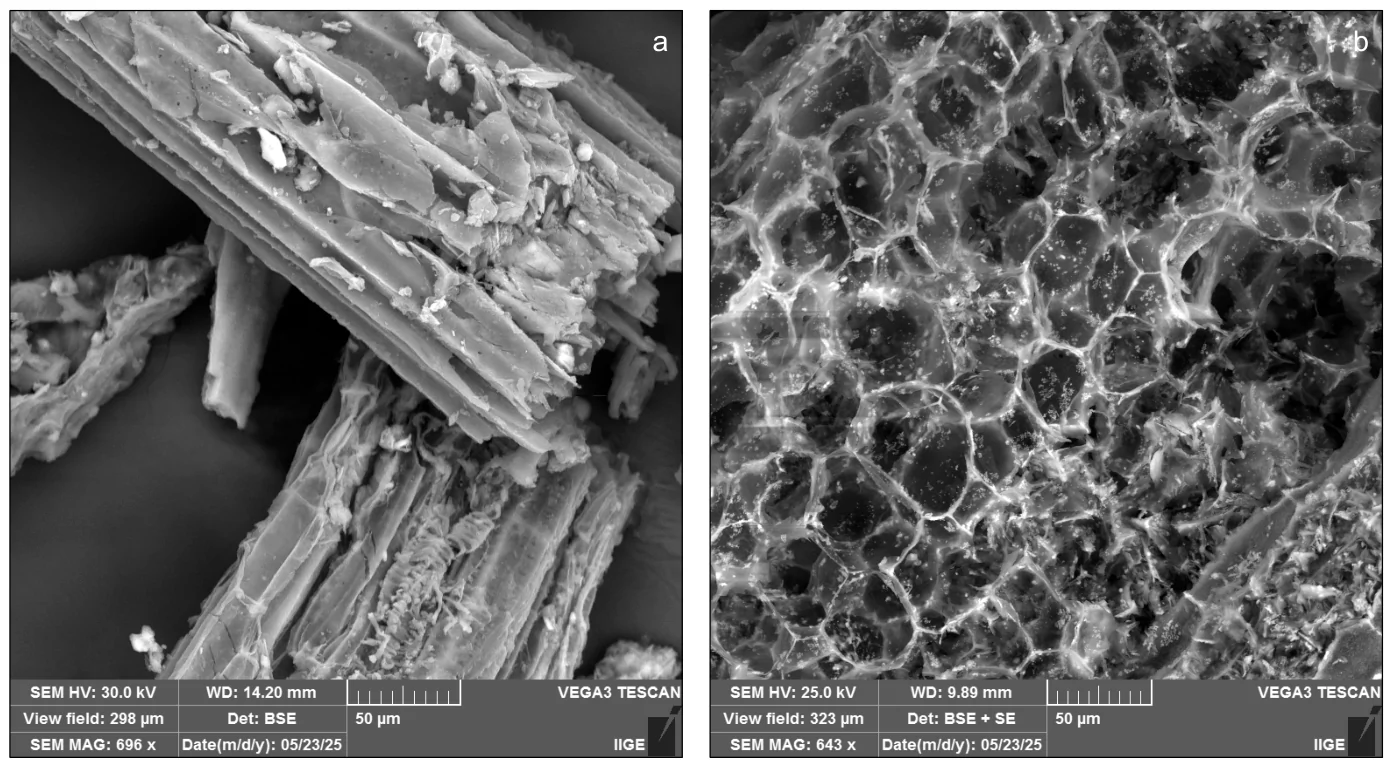
SEM of OFMSW and BC-OFMSW. (**a**) SEM of OFMSW; (**b**) SEM of BC-OFMSW.

SEM analysis was employed to examine the morphological structure of OFMSW and BC-OFMSW. As shown in Figure 5, OFMSW displays a dense, compact surface with negligible visible porosity, which may hinder access to internal adsorption sites. In contrast, BC-OFMSW exhibits a porous and fragmented surface. These morphological changes are consistent with the thermal degradation and volatilization of organic constituents—cellulose, hemicellulose, and lignin—during pyrolysis and improve accessibility to the internal pore network, thereby increasing the available surface area.

### 2.9. Surface Area (BET) and Pore Size Distribution (PSD) of BC-OFMSW and BCA-OFMSW

The textural properties of BC-OFMSW and BCA-OFMSW determined by multipoint BET and NLDFT analyses are summarized in Table 5. For BC-OFMSW, the surface area was 4.40 m^2^ g^−1^ by BET and 4.27 m^2^ g^−1^ by NLDFT, with a pore volume of 0.0085 cm^3^ g^−1^ and an average pore width of 4.887 nm. For BCA-OFMSW, the BET surface area was 786.70 m^2^ g^−1^ and the NLDFT surface area was 1159.58 m^2^ g^−1^, with a pore volume of 0.5045 cm^3^ g^−1^ and an average pore width of 1.868 nm. The BET value of BCA-OFMSW is indicative only, as the BET consistency criteria (Rouquerol) were not satisfied with this sample; the NLDFT value was therefore adopted as the reference surface area.

**Figure 6:**
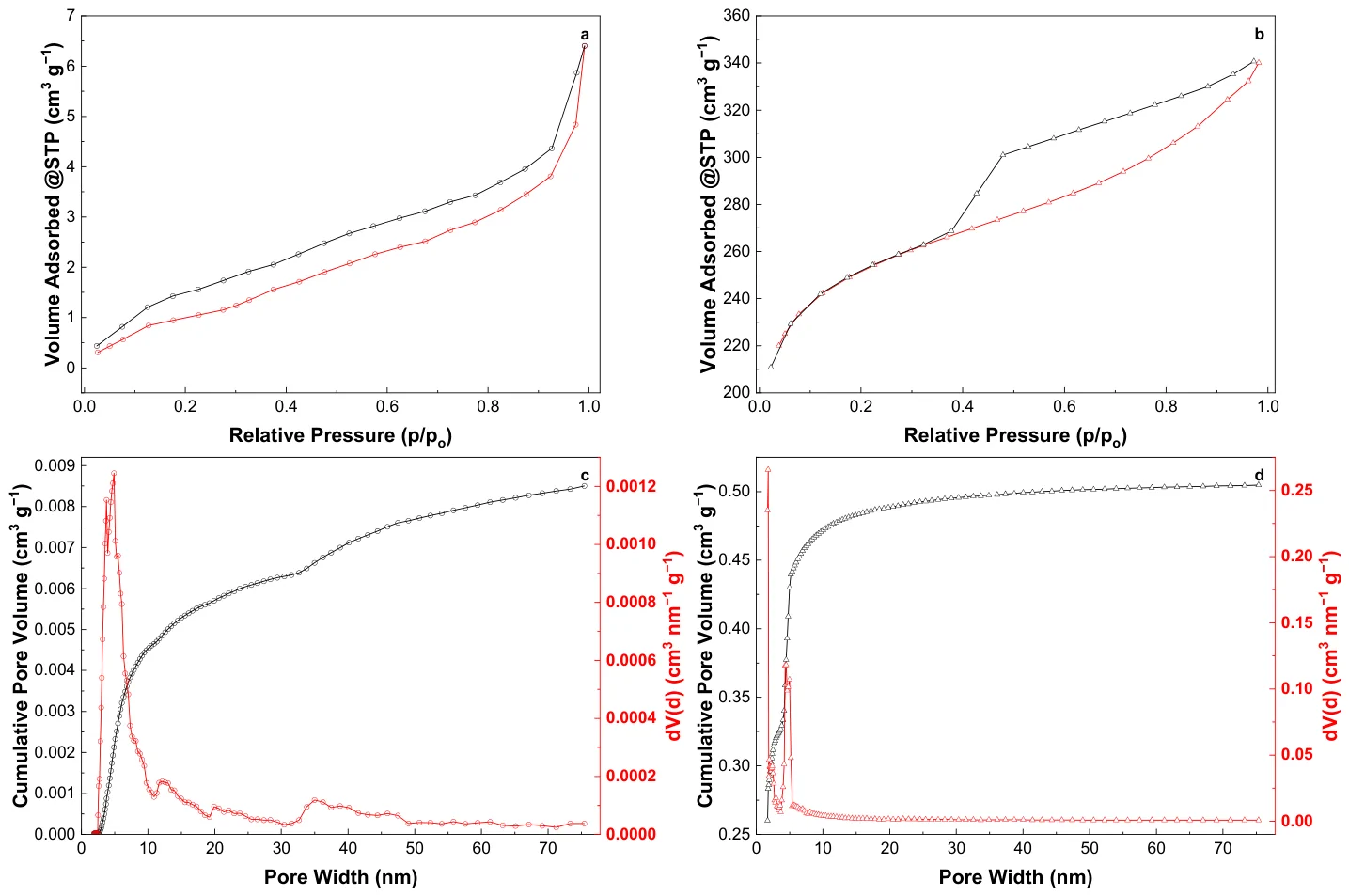
N_2_ adsorption–desorption characterization of BC-OFMSW and BCA-OFMSW at 77 K. (**a**) Linear BET isotherm for BC-OFMSW; (**b**) linear BET isotherm for BCA-OFMSW; (**c**) NLDFT pore volume distribution for BC-OFMSW; (**d**) NLDFT pore volume distribution for BCA-OFMSW.

The N_2_ adsorption–desorption isotherms of both materials are shown in Figure 6a,b, and the NLDFT pore size distributions in Figure 6c,d. The isotherm of BCA-OFMSW shows a steep uptake at low relative pressures followed by a gradual increase, a combination of Type I and Type IV character consistent with the coexistence of micropores and mesopores [31,32]. The adsorption and desorption branches coincide up to P/P_0_ ≈ 0.45 and separate above it, forming an H4-type hysteresis loop. In BC-OFMSW, the N_2_ uptake is very low (below 7 cm^3^ g^−1^) and the two branches do not fully coincide.

The pore size distribution of BC-OFMSW shows a main maximum of about 4–5 nm and a minor contribution near 35 nm (Figure 6c). Such low porosity has been attributed to ash filling or blocking the pores of biochars from the organic fraction of municipal solid waste and from food waste [29,30], which is consistent with the high ash content of BC-OFMSW (37.43 ± 0.53%). After CO_2_ activation, the pore volume increased about 60-fold and most of it is concentrated in narrow pores below ~5 nm, with almost no volume added above ~10 nm (Figure 6d). A decrease in average pore width accompanying an increase in surface area has also been reported for modified biochars [28], and an average pore width of 2.64 nm was reported for an activated carbon from municipal organic solid waste [31]. According to the IUPAC classification, pores below 2 nm are micropores and those between 2 and 50 nm are mesopores [32]; the average pore width of BCA-OFMSW (1.868 nm) therefore falls in the microporous range.

SEM micrographs of BC-OFMSW, the precursor of BCA-OFMSW, reveal a porous, fragmented surface with macropores and cavities in the 1–10 µm range (Figure 5b), consistent with the volatilization of organic constituents during pyrolysis. SEM images of BCA-OFMSW were obtained together with the EDS analysis (Figure 16).

### 2.10. pH, Conductivity and Point of Zero Charge (pH_p_zc)

**Figure 7:**
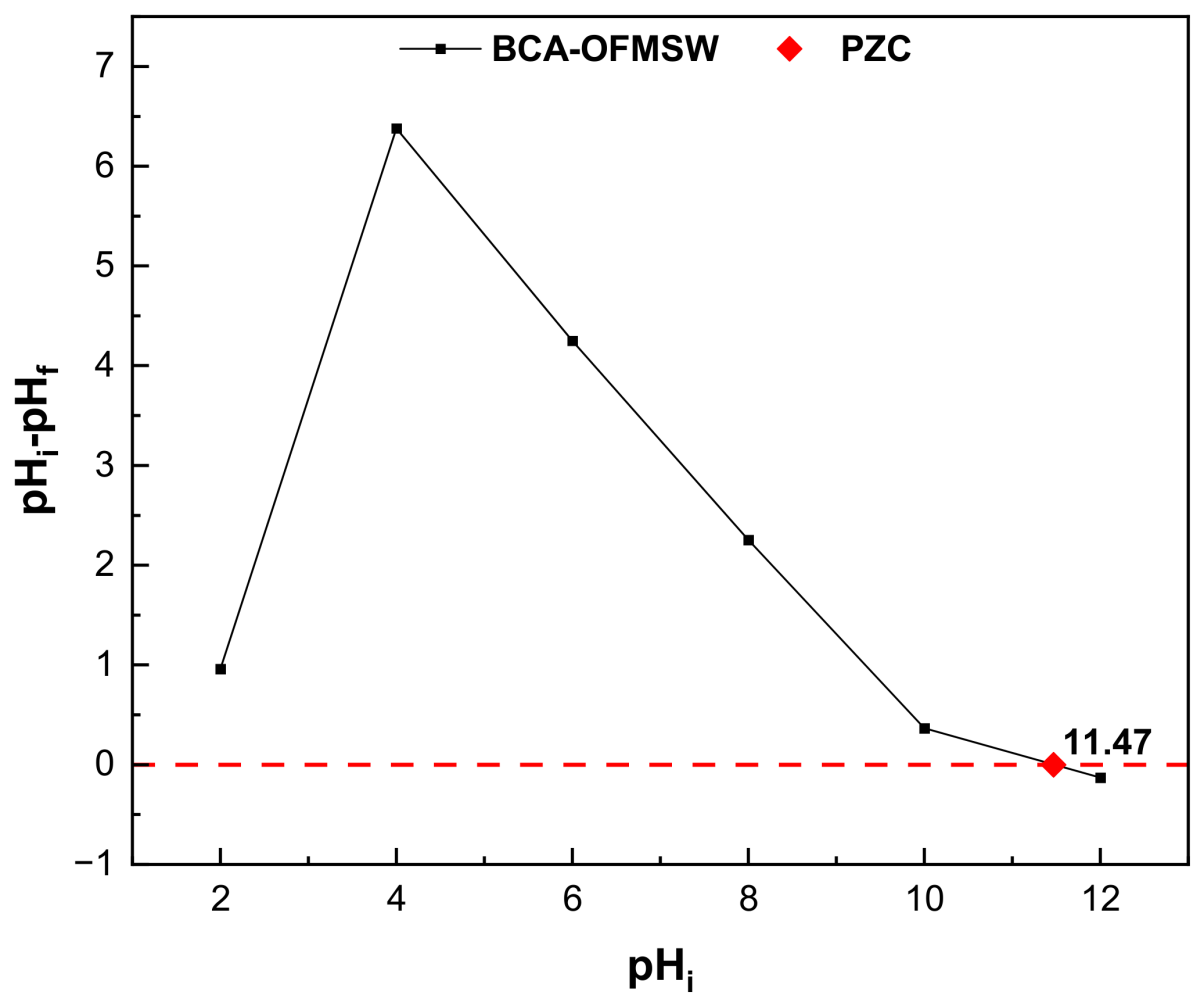
Variation of ΔpH (pH_f_ − pH_i_) as a function of initial pH (pH_i_) for the determination of the point of zero charge (pH_PZC_) of BCA-OFMSW using the pH drift method.

The point of zero charge of BCA-OFMSW was determined by the pH drift method, and the results are shown in Figure 7.

The ΔpH values decreased progressively as the initial pH increased. Positive ΔpH values were observed over most of the pH range evaluated. The intersection between the experimental curve and ΔpH = 0 occurred at pH 11.47, which was identified as the material’s pH_PZC_.

The activated biochar had an average pH of 9.86 ± 0.04 and an electrical conductivity of 536 ± 16 μS cm^−1^ based on triplicate measurements.

### 2.11. Effect of Operational Parameters on the Adsorption Process

#### 2.11.1. Effect of pH

The highest Cr(VI) removal efficiency and equilibrium adsorption capacity were obtained at pH 2, reaching 28.64 ± 1.30% and 55.81 ± 1.61 mg g^−1^, respectively. As the initial pH increased from 2 to 6, both response variables decreased progressively.

**Figure 8:**
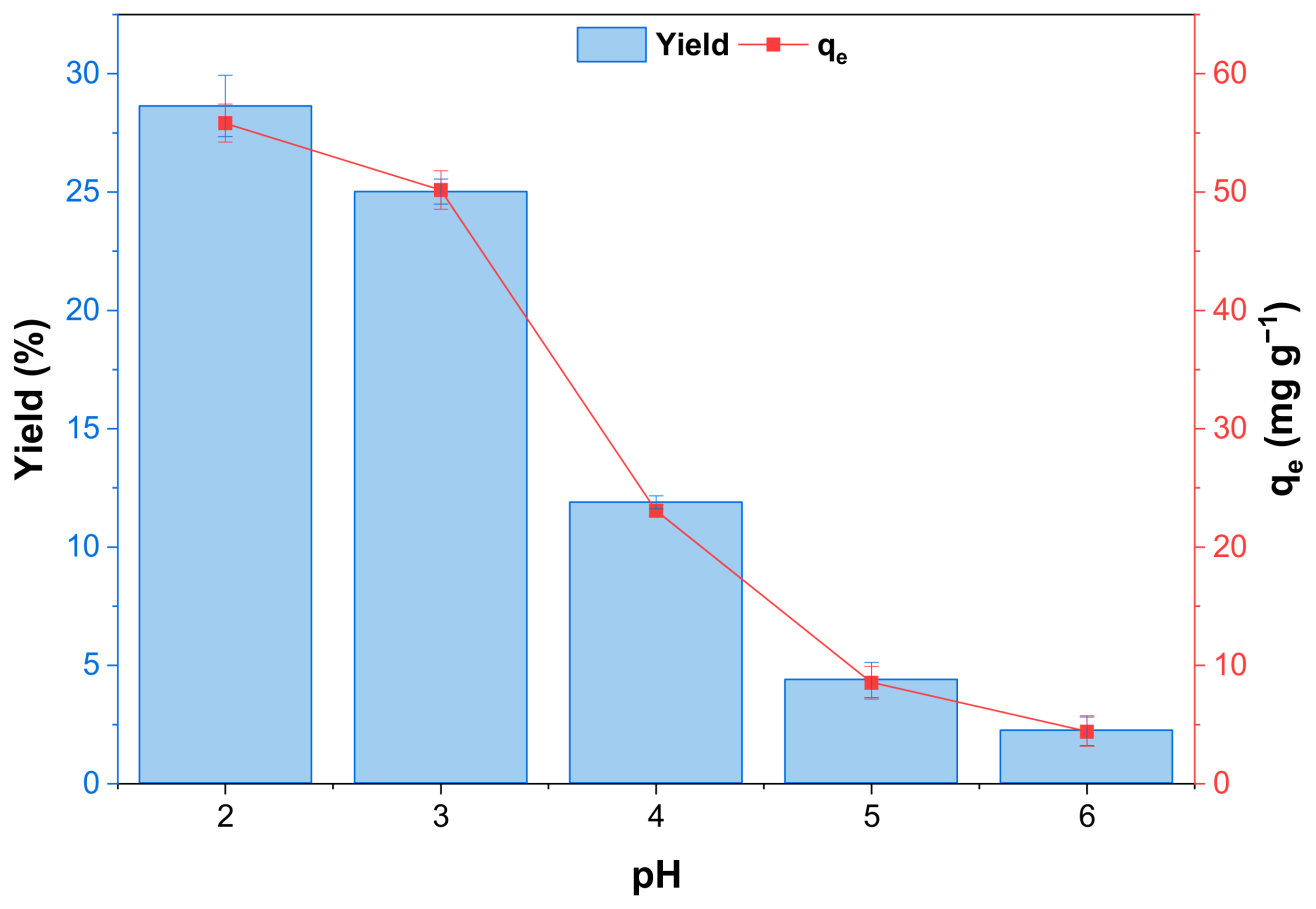
Effect of initial solution pH on Cr(VI) removal efficiency (%) and equilibrium adsorption capacity (q_e_, mg g^−1^) of BCA-OFMSW over the pH range 2–6.

The lowest removal efficiency and adsorption capacity values were recorded at pH 6. The variation of Cr(VI) removal efficiency and equilibrium adsorption capacity as a function of pH is shown in Figure 8.

The decrease in Cr(VI) removal as the initial pH increased from 2 to 6 is consistent with the surface charge of the adsorbent and the speciation of Cr(VI). Below the pH_PZC_, the biochar surface is positively charged and attracts anionic Cr(VI) species such as HCrO_4_^−^ by electrostatic attraction, whereas at higher pH the deprotonation of the surface and the competition of hydroxyl ions for the binding sites hinder Cr(VI) retention [33]. The pH_PZC_ of BCA-OFMSW (11.47) is higher than all the pH values tested, so a net positive surface charge is expected throughout the range, and the lower uptake at higher pH therefore points to a weakening of the electrostatic attraction. Two limitations apply: pH 2 was the lowest value evaluated, so an optimum at or below this value cannot be excluded, and, because the biochar is alkaline, the equilibrium pH may differ from the initial pH, which is the only value reported. The reduction of Cr(VI) to Cr(III) in acidic media, demonstrated by XPS for other biochars [18,33], was not evaluated here and cannot be inferred from the pH dependence.

#### 2.11.2. Effect of Contact Time

Both removal efficiency and adsorption capacity increased with increasing contact time over the interval evaluated. Adsorption increased rapidly during the initial stage of the experiment, followed by a slower increase at longer contact times.

At 120 min, the adsorption capacity reached 61.30 ± 3.15 mg g^−1^, corresponding to a removal efficiency of 31.35 ± 0.51%. Small variations in both parameters were observed between 120 and 240 min. The value at 120 min differs slightly from that of the pH study at pH 2 (55.81 ± 1.61 mg g^−1^; 28.64 ± 1.30%), obtained under identical conditions (200 mg L^−1^ Cr(VI), 50 mg of adsorbent, 30 °C), which reflects the variability between independent experiments.

**Figure 9:**
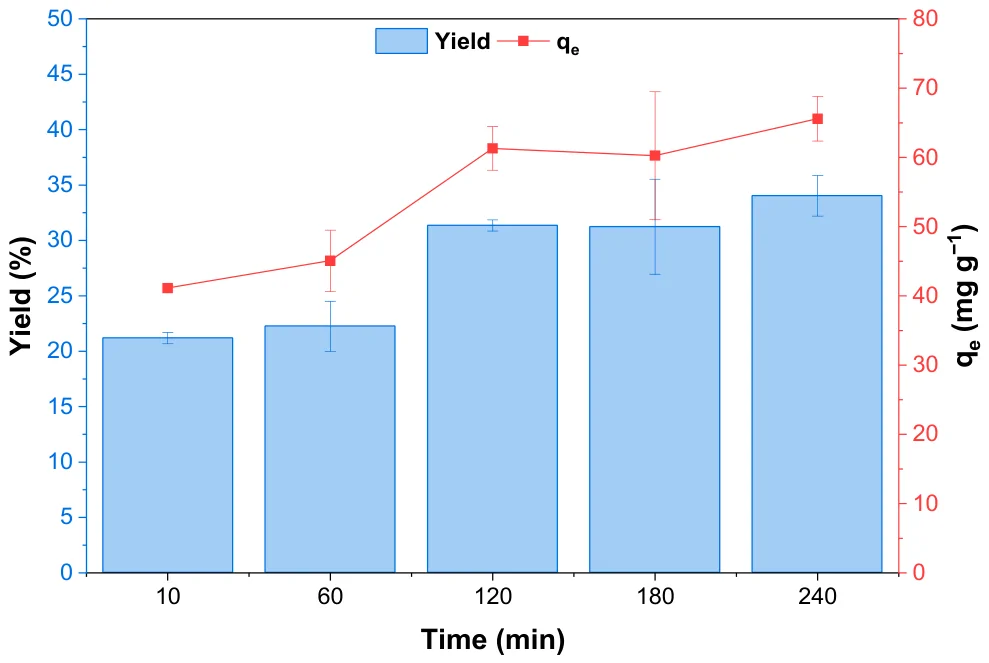
Effect of contact time (t, min) on Cr(VI) removal efficiency (%) and adsorption capacity (q_t_, mg g^−1^) of BCA-OFMSW over the time interval 10–240 min.

Figure 9 shows the temporal evolution of the Cr(VI) removal efficiency and adsorption capacity.

#### 2.11.3. Effect of Adsorbent Dosage

Cr(VI) removal efficiency increased throughout the dosage range evaluated, whereas equilibrium adsorption capacity decreased as adsorbent dosage increased. At a dosage of 100 mg, removal efficiency and adsorption capacity were 43.15 ± 4.40% and 42.13 ± 4.87 mg g^−1^, respectively.

At the highest dosage tested (200 mg), removal efficiency reached approximately 82%, while the corresponding equilibrium adsorption capacity was close to 40 mg g^−1^.

**Figure 10:**
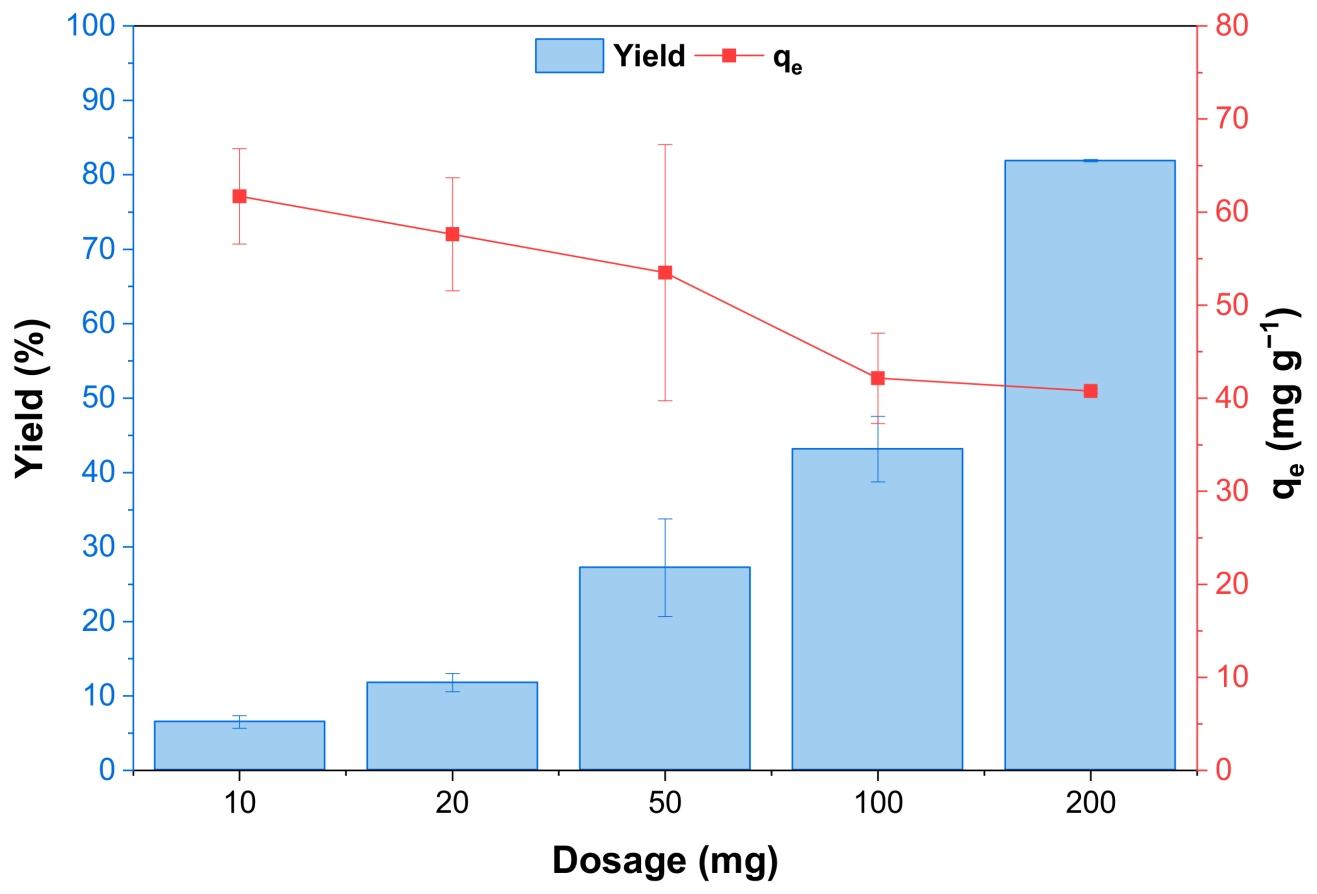
Effect of adsorbent dosage (mg) on Cr(VI) removal efficiency (%) and equilibrium adsorption capacity (q_e_, mg g^−1^) of BCA-OFMSW in batch adsorption experiments.

Figure 10 shows the relationship between adsorbent dosage, removal efficiency, and equilibrium adsorption capacity.

#### 2.11.4. Effect of Initial Concentration Cr(VI)

**Figure 11:**
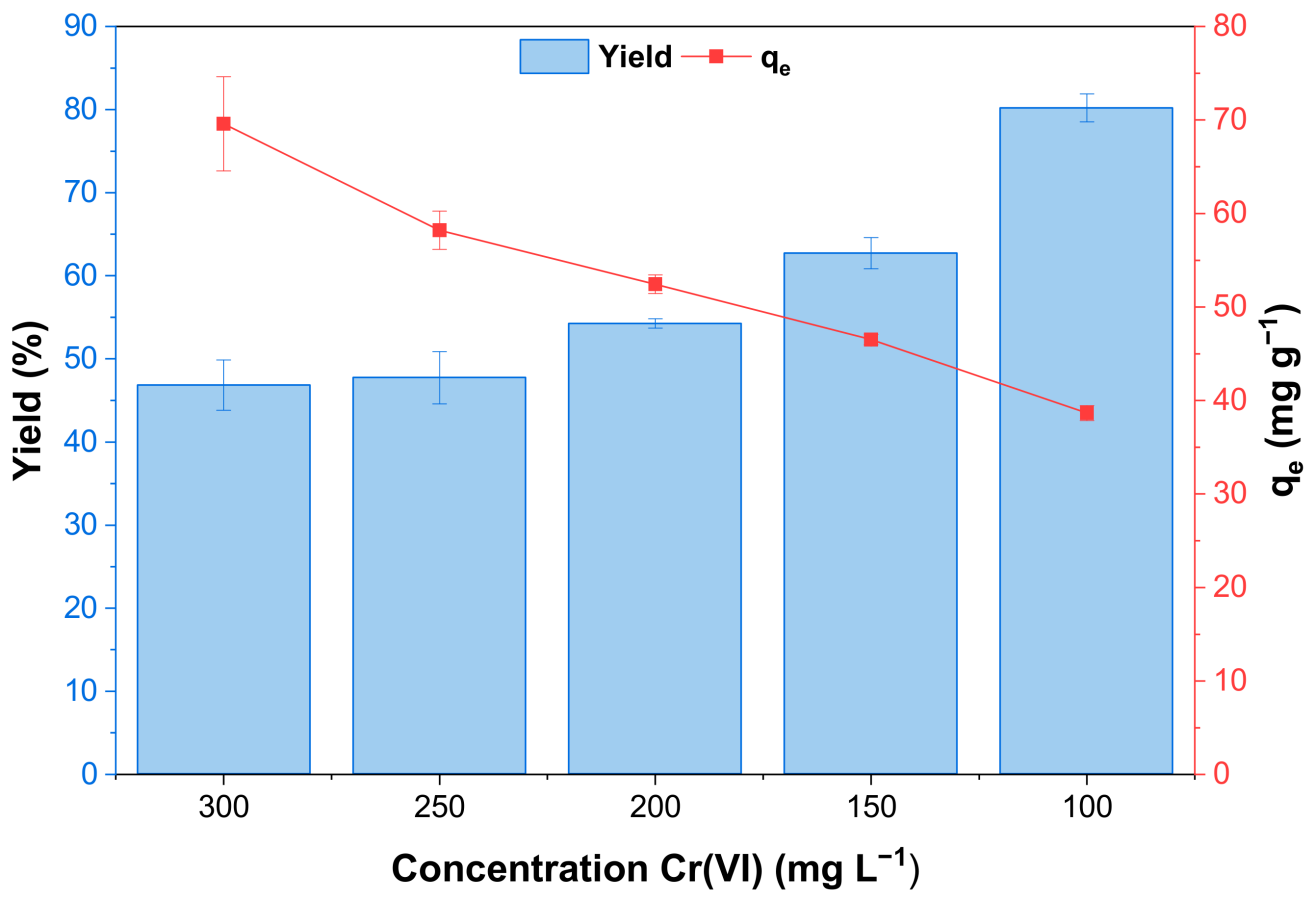
Effect of initial Cr(VI) concentration (mg L^−1^) on Cr(VI) removal efficiency (%) and equilibrium adsorption capacity (q_e_, mg g^−1^) of BCA-OFMSW over the range 100–300 mg L^−1^.

Removal efficiency was highest at 100 mg L^−1^ (80.21 ± 1.69%) and decreased as the initial Cr(VI) concentration increased, whereas the equilibrium adsorption capacity increased with concentration, reaching approximately 69 mg g^−1^ within the 100–300 mg L^−1^ range (Figure 11). Both trends are expected: a larger Cr(VI) load progressively saturates the surface sites and lowers the percentage of removal, while a higher dose provides more accessible binding sites and raises it [30,33,34], as observed in Figure 10 and Figure 11.

These trends also explain why the removal at pH 2 differs between studies: 28.64 ± 1.30% in the pH study (C_0_ = 200 mg L^−1^, 50 mg of adsorbent) and 80.21 ± 1.69% in the concentration study (C_0_ = 100 mg L^−1^, 100 mg of adsorbent). The pH study combined a high concentration with a low dose, two conditions that each limit removal, whereas the concentration study combined a lower concentration with a higher dose. Because of the one-factor-at-a-time (OFAT) design, in which only one variable changed while the others stayed fixed at levels that do not necessarily form a jointly optimized combination [33,35], the removal efficiencies of the four parametric studies cannot be compared directly. The low removal in the pH study therefore reflects the least favorable combination of dose and concentration, and not the effect of pH alone.

The optimum operating conditions identified from the parametric study (pH 2, contact time 120 min, adsorbent dosage 100 mg, and initial Cr(VI) concentration 100 mg L^−1^) were subsequently employed for the adsorption equilibrium, kinetic, and thermodynamic studies.

### 2.12. Adsorption Isotherms

**Figure 12:**
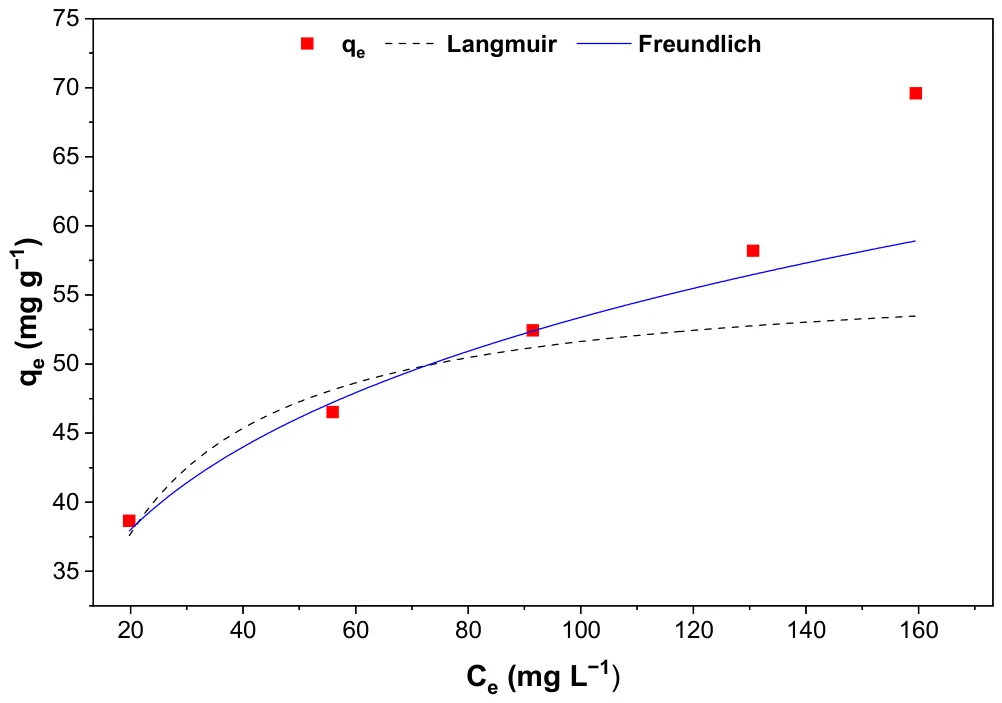
Non-linear fitting of Langmuir and Freundlich isotherm models to the equilibrium Cr(VI) adsorption data (q_e_ vs. C_e_) for BCA-OFMSW.

Equilibrium adsorption data obtained at initial Cr(VI) concentrations of 100–300 mg L^−1^ (pH 2, 100 mg of adsorbent, 120 min; Section 2.11.4) were fitted using the Langmuir and Freundlich isotherm models. Figure 12 presents the fitted curves, and Table 6 summarizes the corresponding parameters. For the Langmuir model, the maximum adsorption capacity (q_m_) was 58.86 ± 3.57 mg g^−1^, and the Langmuir constant (K_L_) was 0.0982 ± 0.0327 L mg^−1^, with a coefficient of determination (R^2^) of 0.8453. For the Freundlich model, the Freundlich constant (K_F_) was 20.16 ± 2.10 mg^1−1/n^ L^1/n^g^-1^, and the heterogeneity factor (1/n) was 0.2114 ± 0.5814, with an R^2^ of 0.9590.

The Freundlich model provided a better fit to the experimental data than the Langmuir model (R^2^ = 0.9590 vs. 0.8453), which suggests that adsorption is described more adequately by an empirical multilayer or heterogeneous-surface approach than by monolayer coverage on uniform sites. The uncertainty of 1/n (±0.5814) exceeds its value, so the Freundlich exponent is poorly constrained. The Langmuir maximum adsorption capacity (58.86 ± 3.57 mg g^−1^) should be interpreted with caution, as this model described the data less well (R^2^ = 0.8453) and the isotherm may not reach a clear plateau within the concentration range studied.

### 2.13. Adsorption Kinetics

**Figure 13:**
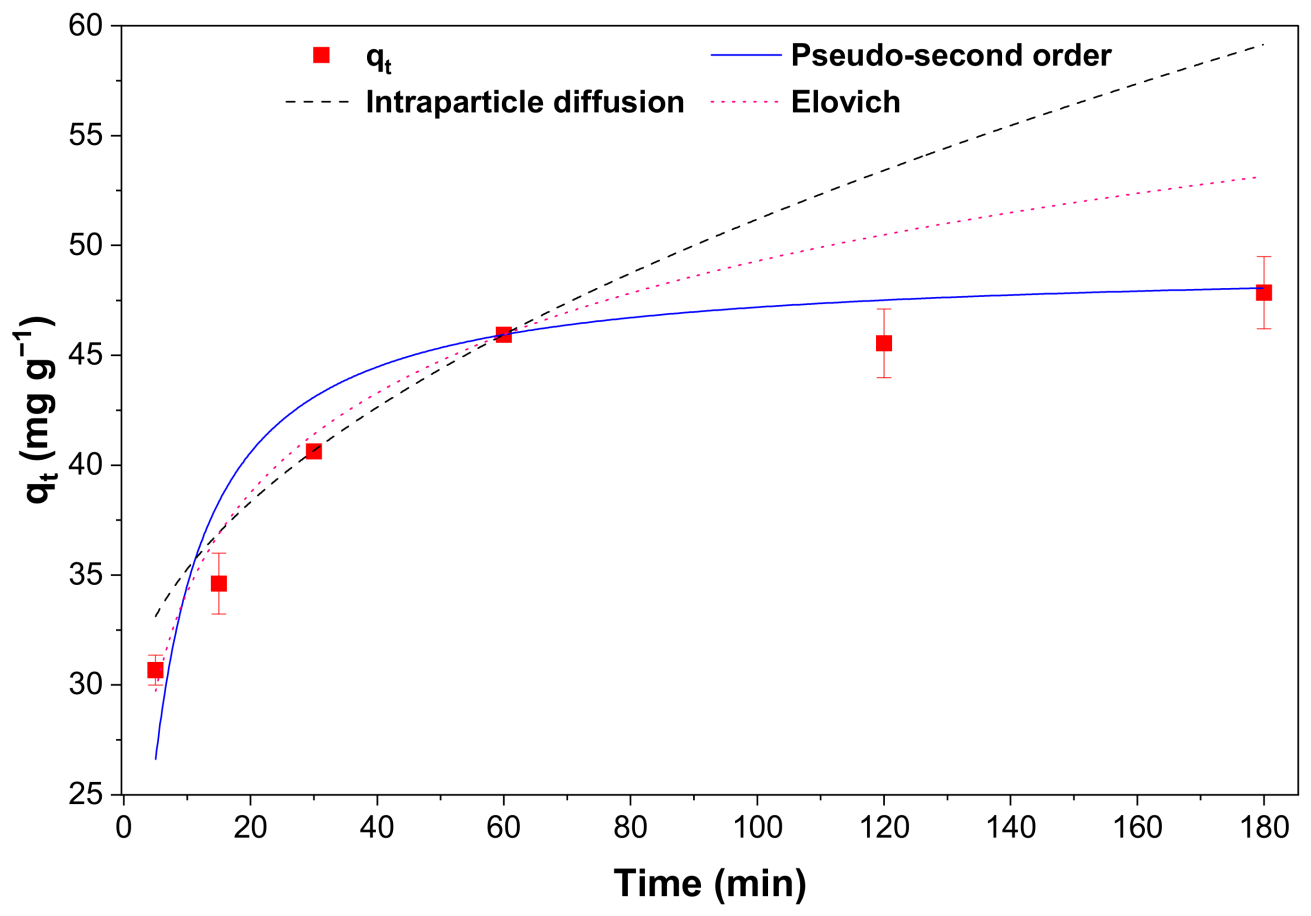
Adsorption kinetics of Cr(VI) onto BCA-OFMSW, showing uptake (q_t_, mg g^−1^) as a function of contact time (t, min) over the interval 0–180 min.

The adsorption kinetics of Cr(VI) onto BCA-OFMSW were evaluated between 5 and 180 min under the selected operational conditions (pH 2, 100 mg of adsorbent, C_0_ = 100 mg L^−1^, and 30 °C). As shown in Figure 13, uptake was rapid during the first 30 min, reaching an adsorption capacity of 40.7 mg g^−1^ (~85% of the capacity observed at 180 min), and leveled off from ~60 min at 46.0–47.9 mg g^−1^. The fast initial uptake is attributed to the steep concentration gradient across the solid–liquid interface and the high initial availability of accessible binding sites [30,33,34].

The experimental kinetic profile was fitted by regression with the pseudo-second-order (PSO), Weber–Morris intraparticle diffusion (ID), and Elovich models (Table 7). By R^2^, the Elovich model gave the best fit (0.9654), followed by the ID (0.9072) and PSO (0.8674) models. However, the PSO model, which has an equilibrium asymptote, reproduced the plateau (k2 = 0.0048 ± 0.0016 g mg^−1^ min^−1^; qe = 49.19 ± 0.99 mg g^−1^), whereas the Elovich and ID models, which have no asymptote, tended to overestimate uptake at extended contact times (Figure 13); R^2^ alone therefore does not establish which model is more appropriate. Although PSO kinetics often prevail in heavy-metal adsorption onto biochars and are commonly interpreted as evidence of chemisorption on homogeneous active sites [30,33,34], the high standard deviation of α in the Elovich model (α = 119.78 ± 69.72 mg g^−1^ min^−1^; β = 0.1524 ± 0.0148 g mg^−1^) indicates that this parameter is poorly constrained by the non-linear regression in this timeframe.

In the Weber–Morris model, the substantial positive intercept (C = 27.89 ± 2.89 mg g^−1^) indicates that intraparticle diffusion is not the sole rate-limiting step, which is consistent with a fast initial uptake at the external surface in addition to diffusion into the micro-mesoporous biochar matrix [30,33,34].

### 2.14. Adsorption Thermodynamics

**Figure 14:**
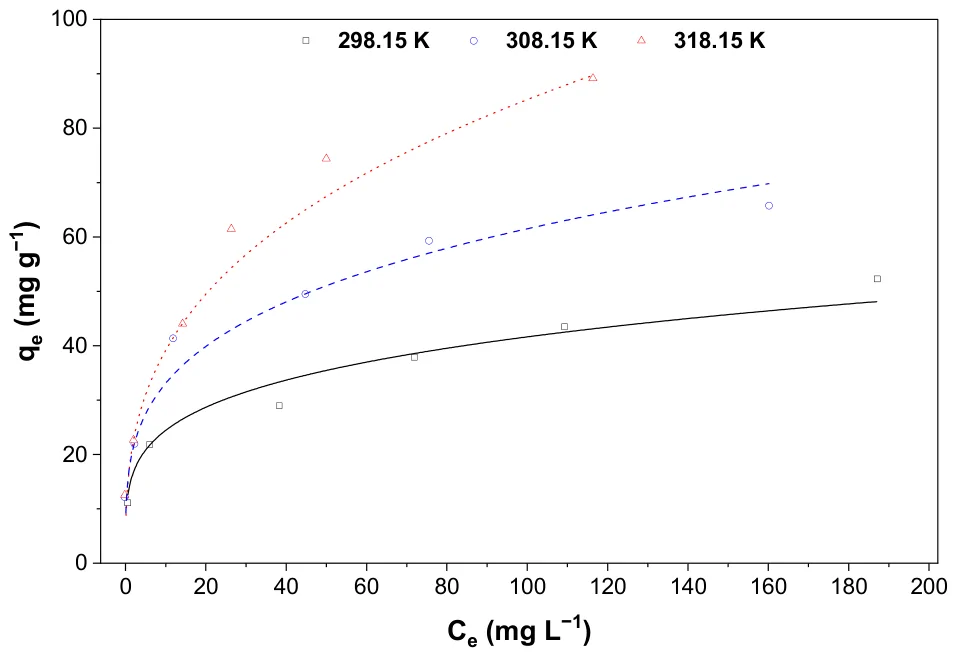
Adsorption isotherms of Cr(VI) onto BCA-OFMSW at 298.15, 308.15, and 318.15 K, fitted with the Freundlich model. Equilibrium capacity (q_e_, mg g^−1^) is plotted as a function of equilibrium concentration (C_e_, mg L^−1^).

The effect of temperature on Cr(VI) adsorption onto BCA-OFMSW was evaluated at 298.15, 308.15, and 318.15 K (Figure 14). Over most of the concentration range, the amount adsorbed increased with temperature, indicating a process favored by heat. To estimate the thermodynamic parameters, the temperature dependence of the Freundlich constant was used (Table 8), yielding ΔH° = +9.08 kJ mol^−1^, ΔS° = +52.84 J mol^−1^ K^−1^, and R^2^ = 0.80 for the Van’t Hoff plot. Because this constant is not a dimensionless equilibrium constant and its use for this purpose has been criticized [33,36], these values are interpreted as an approximate estimate, useful for comparison with other studies rather than as absolute thermodynamic values. The positive sign of ΔH° agrees with the endothermic character suggested by Figure 14, and that of ΔS° may reflect greater randomness at the solid–solution interface [34].

### 2.15. Post-Adsorption Structural Characterization of BCA-Cr

**Figure 15:**
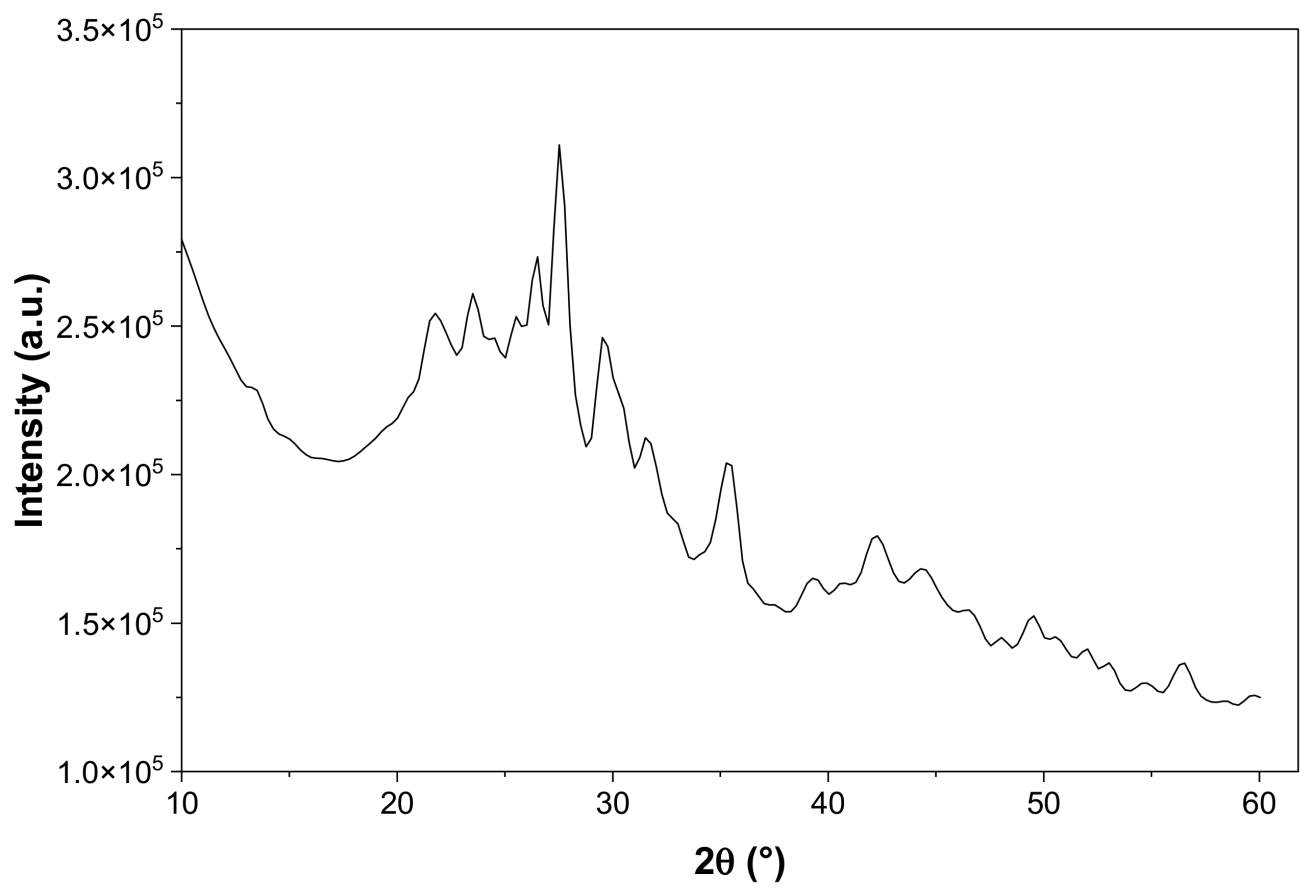
XRD pattern of the post-adsorption biochar (BCA-Cr).

The XRD pattern of the post-adsorption biochar (BCA-Cr) is shown in Figure 15. The diffractogram displayed a broad background signal in the 2θ range of approximately 15–25°, together with several sharp diffraction peaks. The most intense reflection was observed at 2θ = 27.6°, with additional peaks at 21.8°, 23.5°, 26.6°, 29.7°, 35.5°, and 42.4°. The reflection at 26.6° coincides with that assigned to quartz (SiO2) in food-waste biochars before and after adsorption [29]; the remaining reflections, including the most intense one, were not assigned. The sylvite (KCl) reflections observed in BC-OFMSW and BCA-OFMSW (Figure 4) are not evident in BCA-Cr, in agreement with the absence of K and Cl in the EDS analysis (Table 9).

**Figure 16:**
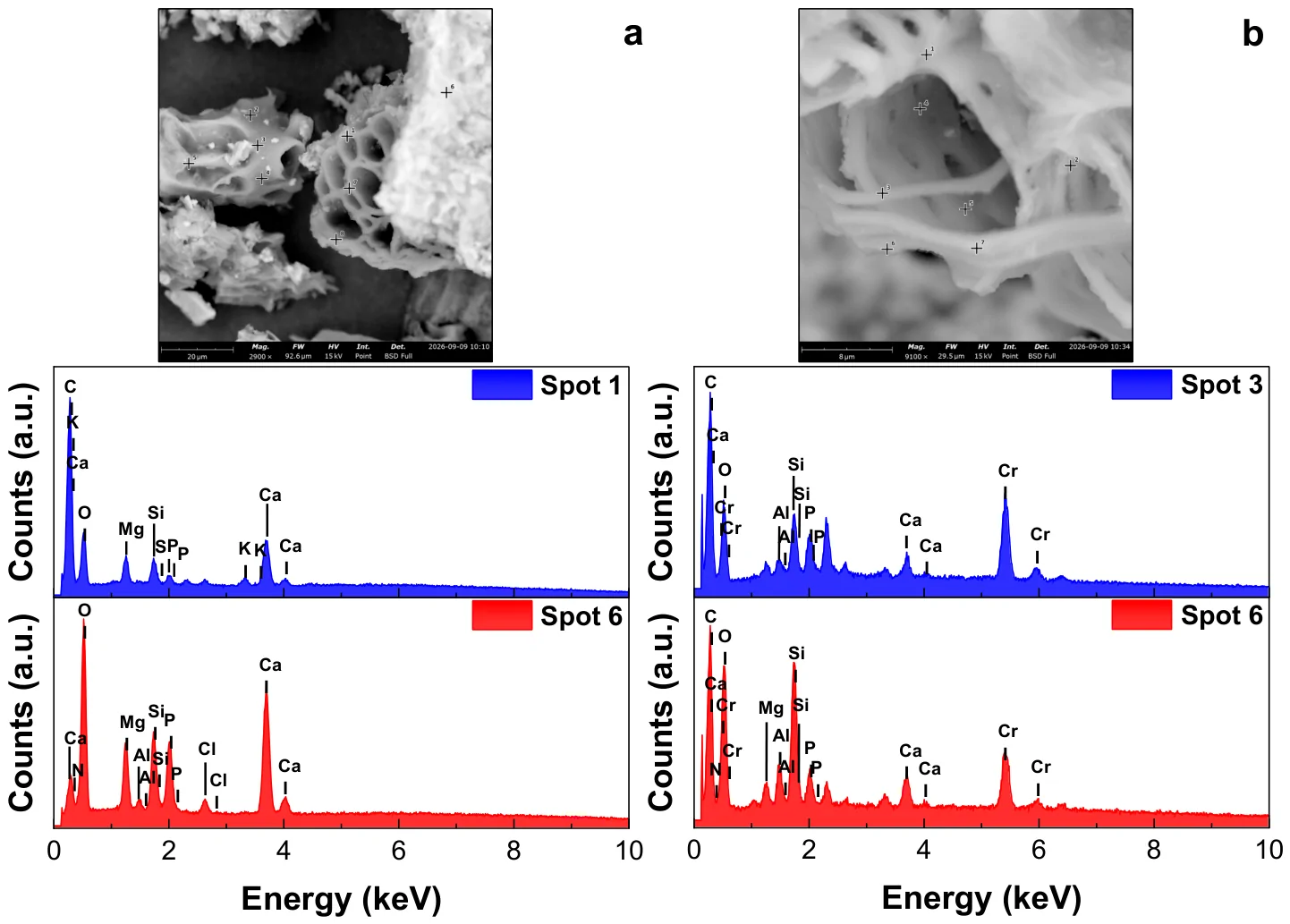
SEM-EDS characterization of BCA-OFMSW and BCA-Cr. (**a**) SEM image and EDS spectra (Spot 1 and Spot 6) of BCA-OFMSW; (**b**) SEM image and EDS spectra (Spot 3 and Spot 6) of BCA-Cr.

The EDS analysis (Table 9, Figure 16) shows chromium at the surface of BCA-Cr (15.07 ± 5.44 wt%), although no reflections were assigned to chromium phases in the XRD pattern. A similar behavior was reported for Cr(III) on a food-waste biochar, where the absence of Cr reflections was interpreted as the absence of crystalline Cr phases on the surface [29]. In the present case, the absence of reflections may also reflect amorphous Cr species or contents below the XRD detection limit, and EDS does not provide the oxidation state of Cr. After adsorption, K and Cl, which were detected in five and three of the eight BCA-OFMSW spots, respectively, were not detected in BCA-Cr. The mean Ca and Mg contents were also lower (Ca from 5.44 ± 5.06 to 1.02 ± 0.77 wt%; Mg from 1.78 ± 1.90 to 0.25 ± 0.44 wt%). The decrease in surface Ca agrees with that reported by Qing et al. [29], who attributed it to the release of Ca to the solution after Cr(III) adsorption. Because EDS is semi-quantitative and the spot-to-spot variability is high, these changes should be read as a trend.

### 2.16. Comparison with Reported Adsorbents

Table 10 compares the maximum Cr(VI) capacities of selected biochar-based adsorbents with BCA-OFMSW. The Langmuir qm of BCA-OFMSW (58.86 mg g^−1^) was obtained at initial concentrations of 100–300 mg L^−1^, 0.1 g of adsorbent, 120 min and pH 2. This value exceeded those of BP–CCAC@ZC3 (19.16 mg g^−1^), BSG AT-5 (31.87 mg g^−1^) and FBCP (12.62 mg g^−1^). The 77.44 mg g^−1^ reported for H-BC (55 °C) is an extrapolated apparent capacity; its highest measured qe was 17.70 mg g^−1^, so no advantage can be established. Conditions differ among studies, so the comparison is indicative. BCA-OFMSW requires no metal reagents, but its preparation (24 h HCl wash, 6 h at 820 °C) is complex, and energy and cost are not reported.

Adsorption capacity did not show a clear relation with surface area among these materials (FBCP, 249 m^2^ g^−1^, 12.62 mg g^−1^; H-BC, 317, 77.44, extrapolated; BP–CCAC@ZC3, 432, 19.16; BCA-OFMSW, 787 by BET and 1160 by NLDFT, 58.86; BSG AT-5, 889, 31.87 mg g^−1^). However, these studies differ in pH, concentration, contact time, and modification, so no trend can be established.

For real effluents (52.3–393.9 mg L^−1^; limit 0.5 mg L^−1^, Introduction), the estimated dose is ≈19–143 g L^−1^ (Langmuir) or ≈3–23 g L^−1^ (Freundlich), versus 2 g L^−1^ in the laboratory tests. Both estimates extrapolate beyond the tested Ce range (19.8–159.5 mg L^−1^) and are indicative. Multistage or column configurations are suggested. Coexisting ions were not evaluated; the reported efficiencies are therefore an upper-bound reference.

## 3. Materials and Methods

### 3.1. Selection and Preparation of OFMSW

For this study, an OFMSW sample was collected from the Manta Wholesale Market in Manta city, Manabí Province, Ecuador (0°57′06.3” S, 80°42′19.7” W). The sampling procedure followed the methodologies outlined in the NOM-AA-15-1985 (“Environmental protection—Soil pollution—Municipal solid waste—Sampling—Quartering method”) [37] (Secretaría de Comercio y Fomento Industrial, 1985) and NTE INEN 2566:2010 (“Aggregates—Reduction of samples to test size”) [38] standards, thereby ensuring representativeness and homogeneity.

Subsequently, the sample was crushed (Model TR-200, TRAPP, Jaraguá do Sul, Brazil) and oven-dried (Model SN30, Memmert, Schwabach, Germany) at 150 °C until the moisture content fell below 10%. After pretreatment, the biomass was transported to the Biomass Laboratory at the Instituto de Investigación Geológico y Energético (IIGE) in Quito, where characterization and experimental procedures were conducted. Sample preparation for the analytical tests followed UNE-EN ISO 14780 [39] and NREL/TP-510-42620 [40] guidelines, ensuring the homogeneity and representativeness of the evaluated portion.

### 3.2. Physicochemical Characterization of OFMSW, BC-OFMSW, BCA-OFMSW

#### 3.2.1. Proximate Analysis, Higher Heating Value, and Ultimate Analysis

The OFMSW samples were characterized via proximate analysis to determine the moisture (%M), volatile matter (%VM), ash (%Ash), and fixed carbon (%FC) contents. Moisture content was evaluated in accordance with ISO 18134-2:2017 [41] using a drying oven (Model SN55, Memmert, Schwabach, Germany) and an analytical balance (Model PA214, OHAUS, Parsippany, NJ, USA). Porcelain crucibles were loaded with approximately 1 g cm^−2^ of sample and cooled in a desiccator for 30 min before each weighing. All determinations were performed in triplicate. In addition, the standard deviation and the coefficient of variation (%cv) were determined.

Volatile matter and ash content determinations were performed following the UNE-EN ISO 18123:2016 [42] and UNE-EN ISO 18122:2016 [43] standards, respectively, employing a muffle furnace (Model F48018, Thermo Scientific, Waltham, MA, USA). In both cases, the crucibles were prepared under the aforementioned conditions, and weighing was conducted after a 60 min desiccation period. The experiment was conducted with three replicates. For BC-OFMSW, the ash content was determined twice. In addition, the standard deviation and the coefficient of variation were determined. The fixed carbon content was calculated by difference using the following equation:

%*FC* = 100−%*M*−%*VM*−%*Ash* (1)

The higher heating value (HHV) was measured in accordance with a methodology based on the UNE-EN ISO 18125 [44] standard, using a bomb calorimeter (Model C2000 basic, IKA^®^, Wilmington, NC, USA). To this end, ~500 mg of the sample was compressed into a compact pellet and then analyzed. The experiment was conducted with three replicates. In addition, the standard deviation and the coefficient of variation were determined.

Finally, the carbon (C), hydrogen (H), nitrogen (N), and sulfur (S) contents of the OFMSW and BC-OFMSW were determined using an elemental analyzer (Model Vario Macro Cube, Elementar, Langenselbold, Germany) via the Dumas combustion method. Samples weighing 10 to 70 mg were placed in tin capsules and introduced into the instrument, which had been previously calibrated with sulfanilamide. The analysis was carried out at 850 °C for both the oxidation and reduction stages. The experiment was conducted with two replicates. In addition, the standard deviation and the coefficient of variation were determined. The evolved gases were analyzed using a thermal conductivity detector (TCD), and the oxygen (O) content was calculated by difference, subtracting the sum of the C, H, N, and S percentages from 100%.

#### 3.2.2. Compositional Analysis

The extractive content of an OFMSW sample was determined in accordance with the procedure described in the NREL/TP-510-42619 [45] method. The experiment was conducted with two replicates. In addition, the standard deviation and the coefficient of variation were determined. In a Soxhlet apparatus, 4.0 g of moisture-free biomass samples were placed in a cellulose thimble (25 × 75 mm) for extraction analysis over a period ranging from 8 to 16 h. The dry biomass was subjected to Soxhlet extraction first with water and then with ethanol; these tests were performed in duplicate. Once the solvent was evaporated, the flask was dried at 50 °C until constant weight was achieved. To determine the extractive content of each sample, a difference calculation was performed by subtracting the mass of the extractive-free biomass from the initial biomass mass. Upon completion of the extraction cycle, the samples recovered from the cellulose thimble were extractive-free and were used to analyze components such as cellulose, hemicellulose, and lignin [31].

The analysis of structural carbohydrates and lignin content in the OFMSW sample was carried out using a method based on the NREL procedure NREL/TP-510-42618, “Determination of Structural Carbohydrates and Lignin in Biomass” [46]. Initially, the sample underwent hydrolysis, after which the amount of structural carbohydrates was determined using a high-performance liquid chromatography (HPLC) system (Model Infinity 1260, Agilent, Santa Clara, CA, USA). The experiment was conducted with two replicates.

#### 3.2.3. FTIR—ATR Spectroscopy

To evaluate the functional groups present in the OFMSW, BC-OFMSW and BCA-OFMSW, Fourier Transform Infrared (FTIR) spectroscopy analysis was performed using a spectrometer (Model FT-IR 460, Jasco, Madrid, Spain) equipped with an Attenuated Total Reflectance (ATR) module. The sample, with a moisture content < 10%, was placed on the instrument’s detection crystal. The spectra were recorded at a resolution of 4 cm^−1^ over an infrared wavenumber range of 600–4000 cm^−1^.

#### 3.2.4. X-Ray Diffraction (XRD)

An X-ray diffractometer (Model Diffractometer D8, Bruker AXS, Karlsruhe, Germany) was used to characterize the OFMSW, BC-OFMSW, BCA-OFMSW, and BCA-Cr. The samples were collected at room temperature, operating at 40 kV and 30 mA, over a 2θ range of 0° to 60° at a scan rate of 0.5°/min, using Cu K-α (1541 Å).

#### 3.2.5. Scanning Electron Microscopy (SEM)

The surface morphology of the OFMSW and BC-OFMSW was characterized by scanning electron microscopy (SEM) using a microscope (Model VEGA3, TESCAN, Brno, Czech Republic). For this purpose, the samples were mounted on stubs with double-sided carbon adhesive tape. Before SEM imaging, the samples were coated with a thin gold film (99.99% purity), approximately 20 nm thick, using a sputter coater [31].

#### 3.2.6. Scanning Electron Microscopy with Energy-Dispersive X-Ray Spectroscopy (SEM-EDS)

The surface morphology and elemental composition of the biochar samples were examined using a Phenom Pro Suite scanning electron microscope (SEM) equipped with energy-dispersive X-ray spectroscopy (EDS). Samples were mounted on aluminum stubs using conductive carbon tape prior to analysis. SEM images were acquired at an accelerating voltage of 10–15 kV, enabling high-resolution observation of surface features.

SEM-EDS point analyses were acquired at eight distinct *spots* for BCA-OFMSW and seven spots for BCA-Cr

#### 3.2.7. Surface Area (BET) and Pore Size Distribution (PSD)

The BET surface area and pore volume analyses of the BC-OFMSW and BCA-OFMSW were performed by nitrogen physisorption at cryogenic temperatures. A chemisorption analyzer (Model AutoChem II 2920, Micromeritics, Norcross, GA, USA) equipped with a thermal conductivity detector (TCD) was employed, following a method based on ISO 9277:2022 [47]. Nitrogen adsorption/desorption was conducted at a 30% nitrogen concentration in helium, using the volume adsorbed under standard temperature and pressure conditions (STP: 0 °C and 760 mmHg).

#### 3.2.8. pH and Conductivity Determination

The pH of the BCA-OFMSW suspensions was measured using a pH meter (Model HQ40D, HACH, Loveland, CO, USA) in accordance with ASTM D1512-15b [48]. For this purpose, the samples were prepared as aqueous suspensions under controlled conditions, allowing the system to stabilize before measurement to ensure the reproducibility and accuracy of the obtained values [32]. The test was performed for triplicates. The electrical conductivity of the same suspensions was also measured and the test was performed three times.

#### 3.2.9. Point of Zero Charge (pH_PZC_)

The point of zero charge (pH_PZC_) of the BCA-OFMSW was determined using a pH meter (Model HQ40D, HACH, Loveland, CO, USA) following the procedure described below. First, 0.1 M KCl solutions were prepared in 50 mL flasks, and their initial pH values were adjusted to 2, 4, 6, 8, 10, and 12 using 0.1 M NaOH and HCl solutions. Once the initial pH stabilized, 0.2 g of BCA-OFMSW was added to each flask, and the mixtures were agitated at 300 rpm for 48 h. After equilibrium, the final pH of each solution was measured. The pH_PZC_ was determined by plotting ΔpH (pH_final_–pH_initial_) as a function of pH_initial_. The pH_PZC_ was obtained at the point where the curve intersects the ΔpH = 0 axis, which corresponds to the pH at which the biochar surface exhibits a net zero charge [32]. The test was measured at ten points and performed twice.

### 3.3. Thermogravimetric Analysis and Derivative Thermogravimetric Analysis

Thermogravimetric analysis (TGA) and derivative thermogravimetric analysis (DTG) were conducted to evaluate the thermal behavior of OFMSW at different heating rates. To this end, samples with particle sizes between 38 and 180 µm and moisture content below 10% were used, with 8–12 mg of the sample placed in the crucible. The analysis was carried out using a thermogravimetric analyzer (Model Pyris 1, Perkin Elmer, USA) under an inert atmosphere by injecting nitrogen (N_2_) at a constant flow rate of 50 mL min^−1^ up to a temperature of 900 °C, employing heating rates (β) of 10, 20, 35, and 50 °C min^−1^.

#### 3.3.1. Thermogravimetric Kinetics of Pyrolysis

According to [33], the non-isothermal decomposition kinetics of a solid at a defined heating rate (*β* = *dT*/*dt*, K min^−1^) follows Equation (2):

(2) $$\frac{\mathrm{d\alpha}}{\mathrm{dT}}=\frac{A}{\beta }\exp\left(\frac{-R}{E_{a}T}\right)f\left(\alpha \right)$$

where *R* represents the gas constant (8.314 J mol^−1^ K^−1^), *T* is the temperature (K), and the function *f(α)* is based on the conversion fraction, represented as:

(3) $$\alpha =\frac{w_{o}-w_{T}}{w_{o}-w_{f}}$$

where *w_o_*, *w_T_*, and *w_f_* represent the mass of the sample in milligrams at the initial temperature, at a given temperature *T*, and at the final temperature, respectively. For the kinetic analysis, the Kissinger–Akahira–Sunose (KAS) and Flynn–Wall–Ozawa (FWO) isoconversional models were employed to estimate the kinetic parameters, specifically the activation energy (*E_a_*, kJ mol^−1^) and the pre-exponential factor (*A*, s^−1^) of the Arrhenius equation. The study was conducted over a conversion degree (*α*) ranging from 0.2 to 0.7, with increments of 0.05.

#### 3.3.2. Thermodynamic Parameters of Pyrolysis

Furthermore, the activation thermodynamic parameters, such as the enthalpy change (Δ*H*, kJ mol^−1^), the Gibbs free energy change (Δ*G*, kJ mol^−1^), and the entropy change (Δ*S*, J mol^−1^ K^−1^), were determined using Equations (4), (5), and (6), respectively:

∆*H* = *E* − *RT* (4)

(5) $$∆G=E+RT_{m}\mathrm{ln ln}\left(\frac{K_{B}T_{m}}{h\;A}\right)$$

where *K_B_* represents the Boltzmann constant (1.380649 10^−23^ J K^−1^), *T_m_* represents the peak temperature at which mass loss occurs, and *h* represents the Planck constant (6.626 10^−34^ J s).

(6) $$∆S=\frac{∆H-∆G}{T_{m}}$$

### 3.4. Preparation of BC-OFMSW

The OFMSW was subjected to a pyrolysis process in a quartz tubular reactor housed inside a muffle furnace (Model Thermolyne, Thermo Scientific, USA), under the following operating conditions: biomass load, 20 g; nitrogen flow rate, 100 mL min^−1^, controlled by an gas mixer (Model IGM, Geometrics Instruments, Barcelona, Spain); heating rate, 20 °C min^−1^; temperature, 575 °C; residence time, 60 min; particle size < 850 µm; and moisture content, <10%, to maximize biochar production. The obtained samples were designated as BC-OFMSW. The experiment was conducted with six replicates. In addition, the standard deviation and the coefficient of variation were determined.

The yield percentage (%R_BC_) across the six runs for biochar production via pyrolysis was calculated using Equation (7):

(7) $$\%R_{\mathrm{BC}}=\frac{m_{\mathrm{BC}-\mathrm{OFMSW}}}{m_{\mathrm{OFMSW}}}\times 100$$

where *m_BC-OFMSW_* is the mass of the obtained biochar, and *m_OFMSW_* corresponds to the mass of the OFMSW introduced into the pyrolysis reactor.

### 3.5. Preparation of BCA-OFMSW

A literature review was conducted to establish the biochar activation procedure [34,35,36]. The material (BC-OFMSW) was acid-washed with a 15% HCl solution at a biochar-to-acid ratio of 1:5 and agitated at 120 rpm for 24 h at room temperature (Model Shaking Incubator SI-64, HYSC, Seoul, Republic of Korea). After this period, successive washes with Type I water preheated to 90 °C were performed until a neutral pH was reached. The biochar was then dried for 2 h at 120 °C. Finally, the washed material was thermally activated by heating at 5 °C min^−1^ to 820 °C under an N_2_ atmosphere at 100 mL min^−1^. Once the target temperature was reached, the atmosphere was switched to CO_2_ at 100 mL min^−1^, and these conditions were maintained for 6 h to obtain the activated OFMSW biochar, designated as BCA-OFMSW. The experiment was conducted with six replicates. In addition, the standard deviation and the coefficient of variation were determined.

### 3.6. Effect of Operational Parameters on the Adsorption Process

Parameter-effect studies were conducted using BCA-OFMSW in synthetic Cr(VI) solutions prepared with analytical grade potassium dichromate (K_2_Cr_2_O_7_) to evaluate how pH, contact time, adsorbent dosage, and initial contaminant concentration affect the material’s removal capacity. For this purpose, the samples were mechanically agitated in a shaking incubator (Model Shaking Incubator SI-64, HYSC, Seoul, Republic of Korea), and the resulting solutions were analyzed using a UV-Vis spectrophotometer (Model DR6000, HACH, Loveland, CO, USA). All experiments were performed in duplicate. A sequential experimental design was used.

#### 3.6.1. Effect of pH

A 50 mL Cr(VI) solution with an initial concentration of 200 mg L^−1^ was prepared, and the pH was adjusted to 2, 3, 4, 5, and 6 using 0.1 M HCl and NaOH. To each solution, 50 mg of BCA-OFMSW was added, and the mixture was shaken on an orbital shaker at 120 rpm for 120 min at 30 °C.

#### 3.6.2. Effect of Contact Time

A 50 mL volume of a 200 mg L^−1^ Cr(VI) solution, adjusted to pH 2 with 0.1 M HCl, was used. Then, 50 mg of BCA-OFMSW was added, and the mixture was shaken on an orbital shaker at 120 rpm at 30 °C for contact times of 10, 60, 120, 180, and 240 min.

#### 3.6.3. Effect of Adsorbent Dosage

A 50 mL Cr(VI) solution with an initial concentration of 200 mg L^−1^, adjusted to pH 2, was prepared. BCA-OFMSW dosages of 10, 20, 50, 100, and 200 mg were evaluated, and all solutions were shaken on an orbital shaker at 120 rpm and 30 °C for 120 min.

#### 3.6.4. Effect of Initial Cr(VI) Concentration

A 50 mL volume of Cr(VI) solutions with initial concentrations of 100, 150, 200, 250, and 300 mg·L^−1^ was prepared. In all cases, the pH was adjusted to 2, 100 mg of BCA-OFMSW was added, and the mixture was shaken on an orbital shaker at 120 rpm and 30 °C for 120 min.

### 3.7. Adsorption Isotherms

The adsorption isotherm study was conducted under the experimental conditions that produced the highest Cr(VI) removal percentages during the parameter effect evaluation stage. The following equations represent the adsorption isotherm models used:

Langmuir:

(8) $$\frac{C_{e}}{q_{e}}=\frac{1}{q_{m}K_{L}}+\frac{C_{e}}{q_{m}}$$

where *C_e_* is the equilibrium concentration of the adsorbate (mg L^−1^), and *K_L_* is the Langmuir constant related to the adsorption capacity or energy of adsorption [37].

Freundlich:

(9) $$q_{e}=\mathrm{log log}K_{F}+\frac{1}{n}\mathrm{log log}C_{e}$$

where *q_e_* refers to the amount of Cr(VI) adsorbed at equilibrium (mg g^−1^), *K_F_* is the Freundlich constant indicating the adsorption capacity, and 1/*n* is the heterogeneity factor used as a measure of the adsorption linearity deviation [35].

The adsorption isotherm models were fitted using OriginPro 2025b software (OriginLab Corporation, Northampton, MA, USA), employing an iterative process to minimize the σ^2^ value. Iterations continued until parameter convergence was achieved, ensuring the best possible model fit, which was evaluated using the coefficient of determination (*R^2^*).

### 3.8. Adsorption Kinetics and Thermodynamics

The kinetic study was conducted under the experimental conditions that produced the highest Cr(VI) removal percentages during the parameter-effect evaluation stage. Additionally, the kinetic parameters were determined using three kinetic models: the pseudo-second-order (PSO) model, the intraparticle diffusion (*k_i_*) model, and the non-linear Elovich (NLE) model, whose equations are presented below:

PSO:

(10) $$q_{t}=\frac{q_{e}^{2}k_{2}t}{1+q_{e}k_{2}t}$$

where *q_t_* represents the amount of Cr(VI) adsorbed per unit of time, *q_e_* is the equilibrium adsorbate loading on the adsorbent (mg g^−1^), and *k*_2_ is the equilibrium rate constant [37].

ID:

*q_t_* = *k_i_t*^1/2^ + *c* (11)

where *k_i_* represents the intraparticle diffusion rate constant (g^−1^ min^−1/2^), and *c* is the intercept, which indicates the thickness of the boundary layer [37].

NLE:

(12) $$q_{t}=\frac{1}{\beta }\mathrm{Ln}(1+\mathrm{\alpha \beta t})$$

where *q_t_* represents the amount of Cr(VI) adsorbed at time *t*, *α* is the desorption constant, and *β* is the initial adsorption rate [35].

The kinetic models were fitted using OriginPro 2025b software (OriginLab Corporation, Northampton, MA, USA), employing an iterative process to minimize σ^2^. Iterations continued until parameter convergence was achieved, ensuring the best possible model fit, which was evaluated using the coefficient of determination (*R^2^*).

The thermodynamic parameters associated with the adsorption of Cr(VI) were calculated using Equations (13), (14), and (15), respectively:

∆*G*^0^ = −*RTlnK_F_* (13)

∆*G*^0^=∆*H*^0^ − *T*∆*S*^0^ (14)

(15) $$\ln K_{F}=\left(\frac{-∆H^{0}}{R}\right)+\frac{∆S^{0}}{R}$$

where *R* = 8.314 (J mol^−1^ K^−1^) represents the universal gas constant, *T* (K) is the absolute temperature at which adsorption occurs, and *K_F_* represents the Freundlich equilibrium constant. These values were determined graphically from the slope and intercept of the plot of *lnK_F_* versus 1/*T*. The tests were performed at three different temperatures: T_1_ = 298.15 K; T_2_ = 308.15 K; and, T_3_ = 318.15 K. It should be noted that, since *K_F_* is not a true thermodynamic equilibrium constant, the Δ*H*° and Δ*S*° values derived from it are presented as approximate estimates rather than absolute thermodynamic values [36,49].

## 4. Conclusions

The results indicate that thermochemical conversion of OFMSW provides a viable route to producing an activated biochar with potential for Cr(VI) removal from aqueous systems. The isoconversional kinetic analysis indicated a multistep pyrolysis, as shown by the progressive increase in apparent activation energy with conversion (KAS and FWO), which is consistent with the heterogeneous composition of OFMSW.

Pyrolysis at 575 °C followed by CO_2_ activation substantially modified the physicochemical properties of the resulting biochar. Activation generated a highly developed porous structure, with an NLDFT surface area of 1159.58 m^2^ g^−1^ and a pore volume of 0.5045 cm^3^ g^−1^. The higher carbon and lower nitrogen and oxygen contents are consistent with a more carbonized and deoxygenated structure, although the H/C ratio increased after pyrolysis and the elemental values should be interpreted with caution.

BCA-OFMSW exhibited an alkaline surface (pH_PZC_ = 11.47), which is consistent with the higher Cr(VI) uptake under acidic conditions. Adsorption depended strongly on pH, adsorbent dosage, initial Cr(VI) concentration, and contact time. The best condition identified through sequential one-factor-at-a-time screening was pH 2, 100 mg of adsorbent, 120 min of contact time, and an initial Cr(VI) concentration of 100 mg L^−1^. Under these conditions, the material showed favorable Cr(VI) removal, and the equilibrium adsorption capacity increased with increasing initial concentration, reaching approximately 69 mg g^−1^ within the investigated range.

The equilibrium and kinetic results describe Cr(VI) uptake as a process in which the Freundlich model (R^2^ = 0.9590) fitted the data better than the Langmuir model (R^2^ = 0.8453), and the Elovich model gave the highest R^2^ among the kinetic models tested (0.9654). The positive intercept of the intraparticle diffusion model indicates that pore diffusion is not the only rate-limiting step. The Langmuir maximum adsorption capacity of 58.86 ± 3.57 mg g^−1^ provides a comparative benchmark with other waste-derived biochars reported in the literature (Table 10).

The pH-dependent behavior and the surface characteristics suggest that electrostatic attraction between positively charged surface sites and anionic Cr(VI) species contributes to Cr(VI) uptake by BCA-OFMSW, possibly together with other surface interactions. Adsorption increased with temperature (Figure 14), but the available data do not provide direct evidence of the adsorption mechanism or of Cr(VI) reduction to Cr(III). The absence of a crystalline chromium phase by XRD, together with the limitations of the thermodynamic parameters derived from the Freundlich constant, means that any contribution from reduction should be regarded as a proposed mechanism rather than an established one.

Overall, BCA-OFMSW shows that a low-value heterogeneous organic waste stream can be converted into a high-surface-area adsorbent with appreciable Cr(VI) removal capacity, providing a promising pathway for simultaneous waste valorization and aqueous pollution control. Nevertheless, further studies using real industrial effluents, particularly those containing competing ions, together with regeneration and reuse cycles and direct characterization of the chemical state of retained chromium, are required to establish the long-term environmental and economic feasibility of the material and its integration into practical treatment systems.

## Figures and Tables

**Table 1:** Physicochemical characterization of the OFMSW (feedstock).

Category	Parameter	Unit	Value
Proximate analysis	Moisture content	% (w.b.) ^1^	3.4059 ± 0.0549 %cv = 1.6119
	Ash content	%	16.9025 ± 0.0744 %cv = 0.4402
	Volatile matter content	%	68.2791 ± 0.1209 %cv = 0.1771
	Fixed carbon content (by difference)	%	11.4126
Calorific value	Higher heating value (HHV)	MJ kg^−1^	15.7037 ± 0.0776 %cv = 0.4941
Ultimate analysis	Carbon (C)	%	32.5944 ± 0.9494 %cv = 2.9128
	Hydrogen (H)	%	5.3381 ± 0.4596 %cv = 8.6098
	Nitrogen (N)	%	10.7602 ± 0.6477 %cv = 6.0194
	Sulfur (S)	%	0.0627 ± 0.0887 %cv = 141.4673
	Oxygen (O, by difference)	%	51.2445 ± 1.9680
Atomic ratios	H/C	a.u.	1.9513
	O/C	a.u.	1.1802
Compositional analysis	Cellulose	%	26.3478 ± 0.7363 %cv = 2.7945
	Hemicellulose	%	10.4573 ± 0.1220 %cv = 1.1666
	Lignin	%	21.4744 ± 0.4104 %cv = 1.9111
	Extractives in water	%	37.0358 ± 0.6668 %cv = 1.8004
	Extractives in ethanol	%	4.6847 ± 0.1066 %cv = 2.2755

^1^ w.b. = wet basis (as received).

**Table 2:** Thermodynamic parameters for OFMSW pyrolysis obtained from the KAS and FWO isoconversional methods over the conversion range 0.20–0.70.

α	R^2^	Ea kJ mol^−1^	LnA 1/s	ΔH kJ mol^−1^	ΔG kJ mol^−1^	ΔS J (mol K)^−1^
KAS						
0.20	0.9502	153.5756	34.6523	149.0864	174.2278	−46.5646
0.25	0.9798	169.5560	37.1052	164.9266	202.0664	−66.7022
0.30	0.9867	170.6193	36.3893	165.8737	200.4306	−60.5440
0.35	0.9879	172.2909	35.8644	167.4329	200.1448	−55.9854
0.40	0.9930	173.9747	35.3403	169.0009	199.7681	−51.4318
0.45	0.9960	157.4644	31.2246	152.3854	162.7935	−17.0397
0.50	0.9930	173.8122	33.9325	168.6417	193.1466	−39.4055
0.55	0.9999	179.1953	34.4003	173.9430	201.2099	−43.1641
0.60	0.9999	182.9003	34.5298	177.5616	205.8813	−44.1047
0.65	0.9962	198.7799	36.7662	193.3272	234.3308	−62.5223
0.70	0.9596	237.3566	42.4473	231.7093	306.0606	−109.4632
Average	0.9856	179.0477	35.6957	173.9899	207.2782	−54.2661
SD	0.0165	22.7569	2.7673	22.4715	37.3910	22.8307
FWO						
0.20	0.9553	154.5705	33.4096	150.0814	169.6441	−36.2328
0.25	0.9819	170.0331	35.9837	165.4037	197.3514	−57.3775
0.30	0.9881	171.2649	35.5188	166.5193	196.9453	−53.3068
0.35	0.9892	173.0695	35.2096	168.2115	197.7424	−50.5414
0.40	0.9938	174.8868	34.8820	169.9131	198.4010	−47.6219
0.45	0.9983	177.1472	34.7284	172.0682	200.2723	−46.1706
0.50	0.9996	176.6907	34.1374	171.5203	197.0843	−41.1087
0.55	0.9999	180.3632	34.4339	175.1110	202.5545	−43.4437
0.60	0.9999	184.0535	34.6935	178.7148	207.9085	−45.4660
0.65	0.9966	199.3805	36.9469	193.9278	235.9171	−64.0251
0.70	0.9631	236.4552	42.4911	230.8079	305.4068	−109.8276
Average	0.9878	181.6287	35.6759	176.5708	209.9298	−54.1020
SD	0.0154	21.1323	2.4482	20.8256	35.1271	20.0227

**Table 3:** Ultimate analysis of OFMSW and BC-OFMSW on a moisture- and ash-free basis.

Category	Parameter	Unit	Value OFMSW	Value BC—OFMSW
Ultimate analysis	Carbon (C)	%	32.5944 ± 0.9494 %cv = 2.9128	41.7488 ± 5.2685 %cv = 12.6195
	Hydrogen (H)	%	5.3381 ± 0.4596 %cv = 8.6098	8.9655 ± 2.5682 %cv = 28.6454
	Nitrogen (N)	%	10.7602 ± 0.6477 %cv = 6.0194	1.1872 ± 0.0811 %cv = 6.8312
	Sulfur (S)	%	0.0627 ± 0.0887 %cv = 141.4673	0.0000 ± 0.0000
	Oxygen (O, by difference)	%	51.2445 ± 1.9680	48.0985 ± 7.9178
Atomic ratios	H/C	a.u.	1.9513	2.5587
	O/C	a.u.	1.1802	0.8648

**Table 4:** Pyrolysis yield of BC-OFMSW and activation yield of BCA-OFMSW.

Parameter	Unit	Value
Pyrolysis yield	%	33.3853 ± 1.1974
Activation yield	%	30.7486 ± 1.8817

**Table 5:** Textural parameters of BC-OFMSW and BCA-OFMSW derived from multipoint BET and NLDFT analyses (N_2_ adsorption at 77 K).

Parameter	Unit	Method	BC-OFMSW	BCA-OFMSW
Surface area	(m^2^ g^−1^)	Multipoint BET	4.400	786.696 *
Surface area	(m^2^ g^−1^)	NLDFT	4.268	1159.575
Pore volume	(cm^3^ g^−1^)	NLDFT	0.0085	0.5045
Pore width	(nm)	NLDFT	4.887	1.868

* Not representative; BET consistency criteria (Rouquerol) not satisfied for this sample—see text. NLDFT value adopted as the reference surface area.

**Table 6:** Langmuir and Freundlich isotherm parameters for Cr(VI) adsorption onto BCA-OFMSW, obtained by non-linear regression of equilibrium data (q_e_ vs. C_e_).

Isotherm Model	Parameter	Unit	Calculated Value	R^2^
Langmuir	q_m_	mg g^−1^	58.8644 ± 3.5743	0.8453
	K_L_	L mg^−1^	0.0982 ± 0.0327	
Freundlich	K_F_	mg^1−1/n^ L^1/n^ g^-1^	20.1583 ± 2.1022	0.9590
	1/n	a.u.	0.2114 ± 0.5814	

**Table 7:** Kinetic parameters for Cr(VI) adsorption onto BCA-OFMSW obtained from non-linear fitting of the pseudo-second-order, intraparticle diffusion, and Elovich models.

Kinetic Model	Parameter	Unit	Calculated Value	R^2^
Pseudo-second order	k_2_	g mg ^−1^min ^−1^	0.0048 ± 0.0016	0.8674
	q_e_	mg g^−1^	49.19 ± 0.99	
Intraparticle diffusion (Weber-Morris)	k_i_	mg g^−1^ min^−1/2^	2.33 ± 0.37	0.9072
	c	mg g^−1^	27.89 ± 2.89	
Elovich	α	mg g^−1^ min^−1^	119.78 ± 69.72	0.9654
	β	g mg ^−1^	0.1524 ± 0.0148	

**Table 8:** Thermodynamic parameters for Cr(VI) adsorption onto BCA-OFMSW derived from the temperature dependence of the Freundlich constant (K_F_).

Parameter	Unit	Calculated Value
ΔH°	kJ mol^−1^	9.0818
ΔS°	J mol^−1^ K^−1^	52.8420
R^2^ (Van’t Hoff)	a.u.	0.80

**Table 9:** Elemental composition (wt%) of BCA-OFMSW and BCA-Cr, determined by EDS analysis.

	BCA-OFMSW									
Element	%wt									
	Spot1	Spot2	Spot3	Spot4	Spot5	Spot6	Spot7	Spot8	Media	sd
C	59.59	56.47	75.71	80.11	70.47	-	62.86	54.97	57.52	24.95
O	29.75	24.35	19.55	12.87	24.62	57.46	21.61	21.94	26.52	13.40
Ca	5.52	-	1.16	2.27	2.42	13.81	6.48	11.87	5.44	5.06
Mg	2.14	-	1.37	1.74	1.27	6.06	1.64	-	1.78	1.90
Si	1.52	-	0.68	1.00	-	5.16	2.18	2.04	1.57	1.67
N	-	19.19	-	-	-	10.32	-	-	3.69	7.23
P	0.73	-	-	0.58	1.22	5.43	0.92	3.12	1.50	1.87
Cl	-	-	-	-	-	1.09	1.53	2.53	0.64	0.97
Al	-	-	-	-	-	0.67	-	-	0.08	0.24
K	0.75	-	0.86	1.44	-	-	2.77	3.53	1.17	1.34
S	-	-	0.68	-	-	-	-	-	0.09	0.24
**BCA-Cr**										
C	59.17	56.97	53.44	50.46	55.71	39.52	53.47	-	52.68	6.44
O	26.25	19.13	22.97	20.98	24.31	32.97	22.05	-	24.09	4.53
Cr	7.29	18.72	15.86	21.47	15.15	8.15	18.85	-	15.07	5.44
N	-	-	-	-	-	9.37	-	-	1.34	3.54
Si	1.75	2.37	2.94	2.56	2.54	4.79	2.17	-	2.73	0.98
P	1.27	2.81	2.41	2.69	2.30	1.27	2.23	-	2.14	0.63
Mg	0.95	-	-	-	-	0.83	-	-	0.25	0.44
Ca	0.90	-	1.44	1.83	-	1.78	1.22	-	1.02	0.77
Al	0.43	-	0.93	-	-	1.32	-	-	0.38	0.54
S	-	-	-	-	-	-	-	-	0.29	0.76

**Table 10:** Comparison of maximum Cr(VI) adsorption capacities (q_m_) reported for biochar-based adsorbents with this study.

Adsorbent	Precursor Material	Pyrolysis T (°C)	Activation/Modification Method	Surface Area (m^2^ g^−1^)	Pore Volume (cm^3^ g^−1^)	q_m_ (mg g^−1^)	Adsorption Conditions	Product Yield (%)	Synthesis Complexity	Ref.
BP–CCAC@ZC3	Banana peel and corn cob (1:1)	600, 2 h	ZnCl_2_ impregnation (NP:ZnCl_2_ 1:1, 4 h at 75 °C) followed by carbonization and washing with 2M HCl	432.14	N.R.	19.16	pH 2, 20 mg L^−1^, 0.4 g, 35 min	N.R.	Medium-high (impregnation, carbonization, and subsequent acid washing)	[23]
BSG AT-5	Brewery spent grain (BSG)	600, 3 h	H_3_PO_4_ impregnation (50% *w*/*w*, 1:1, 12 h) followed by carbonization 3 h	889	0.34	31.87	pH 2, 40 mg L^−1^, 0.1 g, 420 min	N.R.	Medium (acid impregnation and carbonization)	[24]
H-BC	Wood chips and sawdust	700, 4 h	Oxidative functionalization with H_2_O_2_	317.10	0.33	77.44	pH 6, 106.06 mg L^−1^, 0.5 g, 360 min	N.R.	Medium (single-step oxidative functionalization)	[25]
FBCP	Sesame straw	550, 2 h	Impregnation with FeCl_3_·6H_2_O (solid:liquid 1:20); FBCP adds chlorapatite	249.48	1.45	12.62	pH 5, 5–50 mg L^−1^, 0.05 g, 1440 min	N.R.	Medium-high (pyrolysis + impregnation with FeCl_3_, and a second variant using chlorapatite)	[26]
BCA	Organic Fraction of Municipal Solid Waste	575, 1 h	Acid-washed with 15% HCl (biochar:acid ratio 1:5), 120 rpm, 24 h, room temperature; successive washes with Type I water preheated to 90 °C until neutral pH; dried 2 h at 120 °C; thermally activated at 5 °C min^−1^ to 820 °C under N_2_ (100 mL min^−1^), then switched to CO_2_ (100 mL min^−1^) for 6 h	1159.58	0.50	58.86	pH 2, 100 mg L^−1^, 0.1 g, 120 min	33.39% (pyrolysis, OFMSW → BC-OFMSW); 30.75% (activation step, BC-OFMSW → BCA-OFMSW)	High—combines a chemical demineralization step (24 h acid wash) with a physical activation step requiring a switchable-atmosphere furnace (N_2_→ CO_2_ and an extended high-temperature holding time (6 h at 820 °C	This study

## Data Availability

The data presented in this study are available on request from the corresponding author.

## Supplementary Materials

The following supporting information can be downloaded at: https://www.mdpi.com/article/10.3390/molecules31213912/s1. The supplementary files contain the data supporting the findings of this study, including the raw data of the graphs presented in the article, with the replicates of the experimental assays performed, and all the SEM micrographs obtained. Reference [50] is cited in the Supplementary Materials.

## Abbreviations

OFMSW	Organic Fraction of Municipal Solid Waste
BC-OFMSW	Biochar from Organic Fraction of Municipal Solid Waste
BCA-OFMSW	Activated Biochar from Organic Fraction of Municipal Solid Waste
BCA-Cr	Activated Biochar with Hexavalent Chromium Adsorbed
UN	United Nations
MSW	Municipal Solid Waste
SDGs	Sustainable Development Goals
FTIR-ATR	Fourier-Transform Infrared Spectroscopy with Attenuated Total Reflectance
XRD	X-ray Diffraction
SEM	Scanning Electron Microscopy
BET	Brunauer–Emmett–Teller
PSD	Pore Size Distribution
NLDFT	Non-Local Density Functional Theory
HHV	Higher Heating Value
TGA	Thermogravimetric Analysis
DTG	Derivative Thermogravimetry
KAS	Kissinger–Akahira–Sunose Isoconversional Model
FWO	Flynn–Wall–Ozawa Isoconversional Model
w.b.	Wet basis
d.b.	Dry basis
a.u.	Arbitrary unit
OFAT	One Factor at a Time
IIGE	Instituto de Investigación Geológico y Energético
TCD	Thermal Conductivity Detector
AECID	Agencia Española de Cooperación Internacional para el Desarrollo
