# Supplementary material for: Valorization of the Organic Fraction of Municipal Solid Waste (OFMSW): Biochar Production, Activation, and Characterization for Hexavalent Chromium Adsorption
Source: Molecules. 2026 Oct 29;31(21):3912. doi: 10.3390/molecules31213912 (PMC13765172; doi:10.3390/molecules31213912)
Supplement: Supplementary file 1 [file molecules-31-03912-s001.zip › Data Descriptor.pdf]

Data Descriptor

Valorization of the Organic Fraction of Municipal Solid Waste (OFMSW): Biochar Production, Activation, and Characterization for Hexavalent Chromium Adsorption

Daniel A. Rivadeneira [1], Nilo M. Robles Carrillo [1,2,*], Brigitte Rivilla Cueva [1], Cristhian Velalcázar-Rhea [1] and Michelle Romero [1]

S1. Summary

The organic fraction of municipal solid waste (OFMSW) constitutes a substantial share of urban waste streams and, when landfilled without adequate treatment, contributes to greenhouse gas emissions and soil and water contamination. Thermochemical valorization through pyrolysis offers a route to convert this residual biomass into biochar, a carbon-rich solid material with recognized potential as a low-cost adsorbent for water treatment. Among water pollutants of concern, hexavalent chromium Cr(VI) is a priority contaminant due to its high toxicity and carcinogenicity, commonly discharged from steel manufacturing, petroleum and coal refining, metal and mineral processing, the tanning industry, dye production, and welding operations, alongside other industrial applications [1]. Despite growing interest in biochar-based adsorbents, datasets that combine detailed feedstock and material characterization with systematic Cr(VI) adsorption performance data — particularly for biochars derived from OFMSW — remain scarce in the literature, motivating the generation and public release of the dataset described here.

This dataset comprises experimental data generated from OFMSW collected from Manta Wholesale Market in Manta city, Manabí Province, Ecuador (0°57'06.3" S, 80°42'19.7" W) processed into a biochar (BC-OFMSW, obtained by pyrolysis at biomass load, 20 g; nitrogen flow rate, 100 mL min^{-1}, heating rate, 20 °C min^{-1}; temperature, 575 °C; residence time, 60 min; particle size < 850 μm; and moisture content, <10 % and a chemically and physically activated biochar (BCA-OFMSW, obtained by acid washing with 15% HCl followed by CO_2 physical activation at 820 °C). The dataset includes: (i) physicochemical characterization of OFMSW, comprising proximate, ultimate, and compositional analysis, together with its higher heating value; (ii) non-isothermal thermogravimetric data (TGA/DTG) and pyrolysis kinetic parameters (apparent activation energy and pre-exponential factor) derived using the Kissinger–Akahira–Sunose (KAS) and Flynn–Wall–Ozawa (FWO) isoconversional methods, together with the associated thermodynamic parameters; (iii) textural (N_2 physisorption, BET/NLDFT), structural (XRD), morphological (SEM), and surface-chemistry (FTIR, point of zero charge) characterization of BC-OFMSW and BCA-OFMSW; and (iv) batch adsorption data for Cr(VI) removal using BCA-OFMSW, including the effect of operating parameters (pH, contact time, adsorbent dose, and initial concentration), equilibrium isotherms, adsorption kinetics, adsorption thermodynamics, and post-adsorption material characterization.

This dataset was generated within the framework of the research project "Estudio de la obtención de bio-productos a partir de la pirólisis de la fracción orgánica de los residuos sólidos urbanos (FORSU) para mitigación de impactos ambientales y revalorización de residuos", funded by the Agencia Española de Cooperación Internacional para el

Desarrollo (AECID) under grant Number 2023/SPE/0000400068 and developed by the Instituto de Investigación Geológico y Energético (IIGE), Ecuador.

S2. Data Description

S2.1. Physicochemical Characterization of OFMSW

Table S1. Moisture content data.

Sample	Replicate	%H	Mean	SD
OFMSW	1	3.4590	3.4059	0.0549
	2	3.4093		
	3	3.3493		

Table S2. Ash content data.

Sample	Replicate	%Cz	Mean	SD
OFMSW	1	16.8249	16.9025	0.0744
	2	16.9094		
	3	16.9732		

Table S3. Volatile Matter content data.

Sample	Replicate	%VM	Mean	SD
OFMSW	1	68.1721	68.2791	0.1209
	2	68.2549		
	3	68.4102		

Table S4. Fixed carbon content (calculated by difference).

Sample	%H	%CZ	%VM	%CF
OFMSW	3.4059	16.9025	68.2791	11.4126

Table S5. Higher heating value (HHV) content.

Sample	Replicate	HHV, MJ kg^{-1}	Mean	SD
OFMSW	1	15.632	15.7037	0.0776
	2	15.693		
	3	15.786		

Table S6. Ultimate analysis content data.

Sample	Replicate	%N	%C	%H	%S	%Humidity	%CZ
OFMSW	1	8.21	25.44	3.9950	0.1	3.4059	16.9025
	2	8.94	26.51	4.5130	0		

Sample	%N	SD	%C	SD	%H	SD	%S	SD	%O	SD
OFMSW	10.7602	0.6477	32.5944	0.9494	5.3381	0.4596	0.0627	0.0887	51.2445	1.9680

Table S7. Cellulose, hemicellulose, and lignin contents data.

Sample	Replicate	%Lignin	Media	SD	%Cellulose	Media	SD	%Hemicellulose	Media	SD
OFMSW	1	22.8537	23.2990	0.4453	27.7878	28.5865	0.7989	11.2377	11.3458	0.1324
	2	23.7444			29.3853			11.4540		

Table S8. Extractives in water and extractives in ethanol content data.

Sample	Replicate	%Ext_{water}	Media	SD	%$Ext_{Ethanol}$	Media	SD
OFMSW	1	39.6712	40.1827	0.7234	5.1646	5.0828	0.1157
	2	40.6942			5.0010		

Values were normalized (adjusted to 100%) to correct the initial total sum to report the final relative content of each structural component.

Table S9. Values normalized.

OFMSW	Mean
%Cellulose	26.3478
SD	0.7363
%Hemicellulose	10.4573
SD	0.1220
%Lignin	21.4744
SD	0.4104
%Ext_{water}	37.0358
SD	0.6668
%$Ext_{Ethanol}$	4.6847
SD	0.1066

S2.2. Thermal Behavior of OFMSW

The TGA analysis data is available in the file "DATA_TGA_OFMSW.csv".

The DTG analysis data is available in the file "DATA_DTG_OFMSW.csv".

S2.3. Thermogravimetric Kinetics of Pyrolysis

Table S10. KAS method data.

α	T^{-1}	$Ln(\beta/T^2)$	T^{-1}	$Ln(\beta/T^2)$	T^{-1}	$Ln(\beta/T^2)$	T^{-1}	$Ln(\beta/T^2)$
	K^{-1}		K^{-1}		K^{-1}		K^{-1}	
		10°C min^{-1}		20°C min^{-1}		35°C min^{-1}		50°C min^{-1}
0.0200	0.0030	-9.3379	0.0030	-8.6219	0.0028	-8.2353	0.0029	-7.7841
0.0500	0.0027	-9.5284	0.0027	-8.8086	0.0025	-8.4641	0.0026	-8.0021
0.1000	0.0021	-9.9999	0.0022	-9.2411	0.0020	-8.8599	0.0021	-8.4152
0.1500	0.0020	-10.1513	0.0020	-9.4718	0.0019	-8.9821	0.0019	-8.6183
0.2000	0.0019	-10.2381	0.0019	-9.5654	0.0018	-9.0534	0.0018	-8.7081
0.2500	0.0018	-10.3002	0.0018	-9.6312	0.0018	-9.1096	0.0018	-8.7702
0.3000	0.0018	-10.3483	0.0018	-9.6819	0.0017	-9.1578	0.0017	-8.8215
0.3500	0.0017	-10.3944	0.0017	-9.7293	0.0017	-9.2032	0.0017	-8.8698
0.4000	0.0017	-10.4399	0.0017	-9.7777	0.0017	-9.2504	0.0016	-8.9172
0.4500	0.0017	-10.4801	0.0016	-9.8213	0.0016	-9.2929	0.0016	-8.9584
0.5000	0.0016	-10.5138	0.0016	-9.8576	0.0016	-9.3310	0.0016	-8.9931
0.5500	0.0016	-10.5449	0.0016	-9.8901	0.0016	-9.3618	0.0016	-9.0242
0.6000	0.0016	-10.5782	0.0016	-9.9221	0.0015	-9.3940	0.0015	-9.0572
0.6500	0.0016	-10.6243	0.0015	-9.9646	0.0015	-9.4325	0.0015	-9.0994
0.7000	0.0015	-10.7024	0.0015	-10.0370	0.0015	-9.4942	0.0014	-9.1678
0.7500	0.0014	-10.8198	0.0014	-10.1432	0.0014	-9.5909	0.0014	-9.2671
0.8000	0.0013	-10.9619	0.0013	-10.2623	0.0013	-9.7029	0.0013	-9.3796
0.8500	0.0012	-11.1418	0.0012	-10.4041	0.0012	-9.8309	0.0012	-9.5150
0.9000	0.0010	-11.4378	0.0011	-10.7008	0.0011	-10.0583	0.0011	-9.7795

0.9500	0.0009	-11.7251	0.0009	-11.0507	0.0009	-10.4433	0.0009	-10.0949
0.9800	0.0009	-11.8347	0.0009	-11.1398	0.0009	-10.5831	0.0008	-10.2321

Table S11. FWO method data.

α	**T^{-1}**	**Log(β)**	**T^{-1}**	**Log(β)**	**T^{-1}**	**Log(β)**	**T^{-1}**	**Log(β)**
	**K^{-1}**		**K^{-1}**		**K^{-1}**		**K^{-1}**	
		**10°C min^{-1}**		**20°C min^{-1}**		**35°C min^{-1}**		**50°C min^{-1}**
0.0200	0.0030	1.0000	0.0030	1.3010	0.0028	1.5441	0.0029	1.6990
0.0500	0.0027	1.0000	0.0027	1.3010	0.0025	1.5441	0.0026	1.6990
0.1000	0.0021	1.0000	0.0022	1.3010	0.0020	1.5441	0.0021	1.6990
0.1500	0.0020	1.0000	0.0020	1.3010	0.0019	1.5441	0.0019	1.6990
0.2000	0.0019	1.0000	0.0019	1.3010	0.0018	1.5441	0.0018	1.6990
0.2500	0.0018	1.0000	0.0018	1.3010	0.0018	1.5441	0.0018	1.6990
0.3000	0.0018	1.0000	0.0018	1.3010	0.0017	1.5441	0.0017	1.6990
0.3500	0.0017	1.0000	0.0017	1.3010	0.0017	1.5441	0.0017	1.6990
0.4000	0.0017	1.0000	0.0017	1.3010	0.0017	1.5441	0.0016	1.6990
0.4500	0.0017	1.0000	0.0016	1.3010	0.0016	1.5441	0.0016	1.6990
0.5000	0.0016	1.0000	0.0016	1.3010	0.0016	1.5441	0.0016	1.6990
0.5500	0.0016	1.0000	0.0016	1.3010	0.0016	1.5441	0.0016	1.6990
0.6000	0.0016	1.0000	0.0016	1.3010	0.0015	1.5441	0.0015	1.6990
0.6500	0.0016	1.0000	0.0015	1.3010	0.0015	1.5441	0.0015	1.6990
0.7000	0.0015	1.0000	0.0015	1.3010	0.0015	1.5441	0.0014	1.6990
0.7500	0.0014	1.0000	0.0014	1.3010	0.0014	1.5441	0.0014	1.6990
0.8000	0.0013	1.0000	0.0013	1.3010	0.0013	1.5441	0.0013	1.6990
0.8500	0.0012	1.0000	0.0012	1.3010	0.0012	1.5441	0.0012	1.6990
0.9000	0.0010	1.0000	0.0011	1.3010	0.0011	1.5441	0.0011	1.6990
0.9500	0.0009	1.0000	0.0009	1.3010	0.0009	1.5441	0.0009	1.6990
0.9800	0.0009	1.0000	0.0009	1.3010	0.0009	1.5441	0.0008	1.6990

S2.5. Physicochemical Composition of OFMSW and BC-OFMSW and Yield of BC-OFMSW and BCA-OFMSW

Table S12. BC-OFMSW ash content data.

**Sample**	**Replicate**	**%CZ**	**Mean**	**SD**
BC-OFMSW	1	37.8017	37.4286	0.5277
	2	37.0554		

Table S13. BC-OFMSW ultimate analysis content data.

**Sample**	**Replicate**	**%N**	**%C**	**%H**	**%S**	**%Humidity**	**%CZ**
BC-OFMSW	1	0.76	27.77	6.584	0	1.5038	37.4286
	2	0.69	23.22	4.366	0		

**Sample**	**%N**	**SD**	**%C**	**SD**	**%H**	**SD**	**%S**	**SD**	**%O**	**SD**
BC-OFMSW	1.1872	0.0811	41.7488	5.2685	8.9655	2.5682	0.0000	0.0000	48.0985	7.9178

Table S14. Pyrolysis yield of BC-OFMSW and activation yield of BCA-OFMSW content data.

Sample	Replicate	Yield, %	Mean	SD
BC-OFMSW	1	32.6073	33.3853	1.1974
	2	32.8369		
	3	34.2433		
	4	31.6345		
	5	34.4951		
	6	34.4944		
BCA-OFMSW	1	29.4180	30.7486	1.8817
	2	32.0791		
	3	33.3247		
	4	28.2698		
	5	31.5300		
	6	29.8700		

S2.6. Surface chemistry: FTIR-ATR of OFMSW, BC-OFMSW and BCA-OFMSW

The FTIR-ATR analysis of OFMSW data is available in the file "DATA_FTIR_OFMSW.csv".

The FTIR-ATR analysis of BC-OFMSW data is available in the file "DATA_FTIR_BC-OFMSW.csv".

The FTIR-ATR analysis of BCA-OFMSW data is available in the file "DATA_FTIR_BCA-OFMSW.csv".

S2.7. Structural Characterization (XRD) of OFMSW, BC–OFMSW and BCA–OFMSW

The XRD analysis of OFMSW data is available in the file "DATA_DRX_OFMSW.csv"

The XRD analysis of BC-OFMSW data is available in the file "DATA_DRX_BC-OFMSW.csv"

The XRD analysis of BCA-OFMSW data is available in the file "DATA_DRX_BCA-OFMSW.csv"

S2.8. Morphological Characterization (SEM) of OFMSW and BC-OFMSW

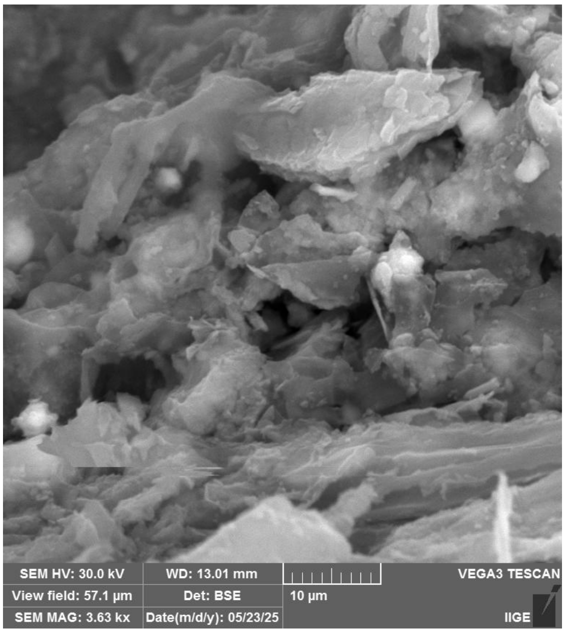

Figure S1. SEM of OFMSW at 10 μm.

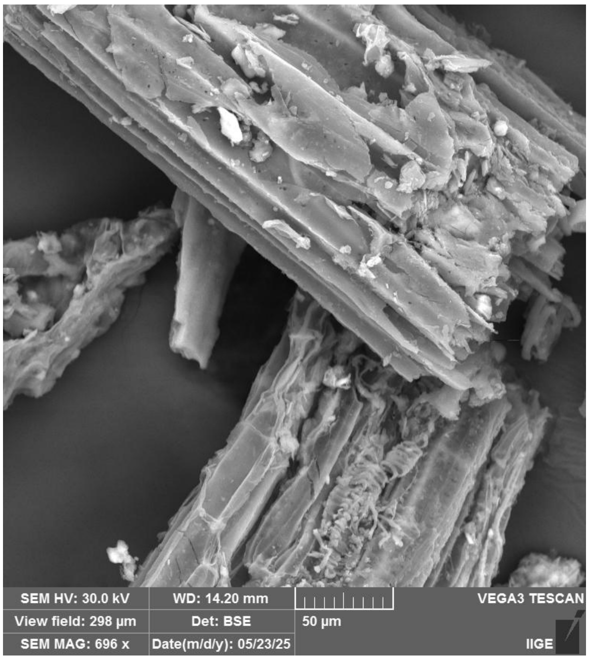

Figure S2. SEM of OFMSW at 50 μm.

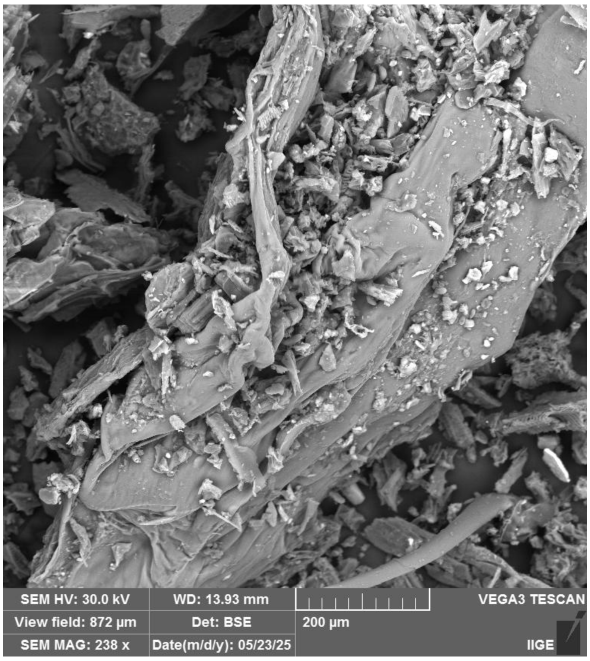

Figure S3. SEM of OFMSW at 200 μm.

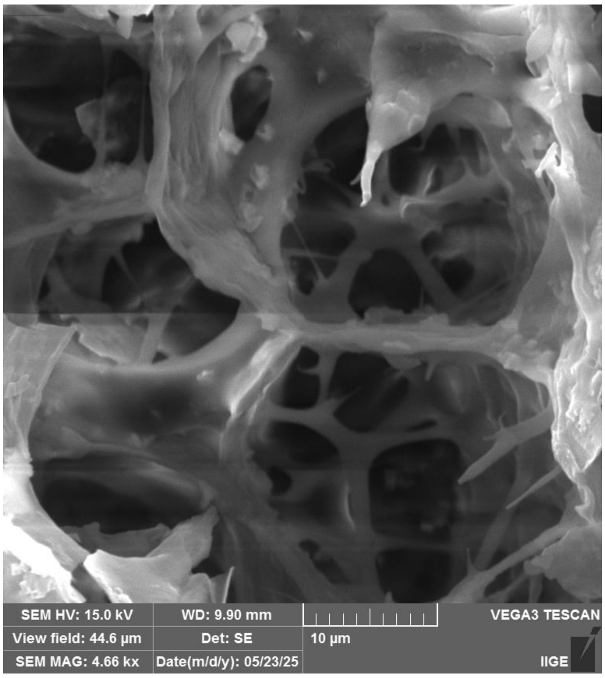

Figure S4. SEM of BC-OFMSW at 10 μm.

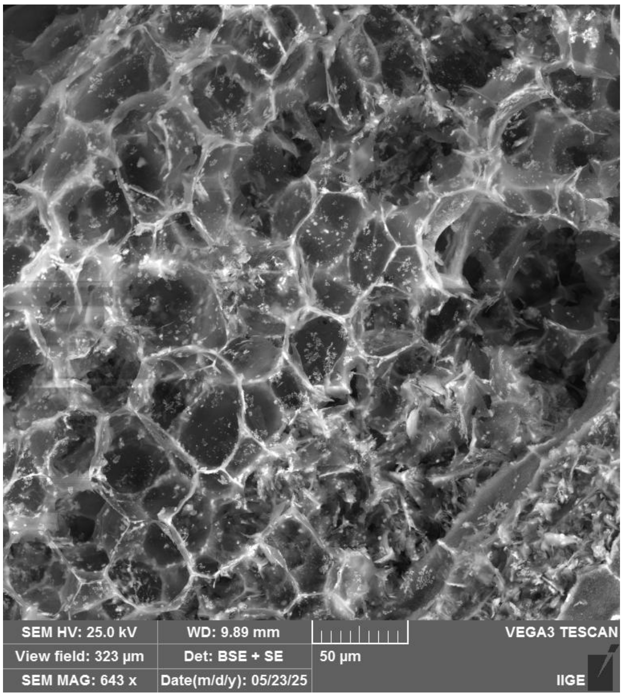

Figure S5. SEM of BC-OFMSW at 50 μm.

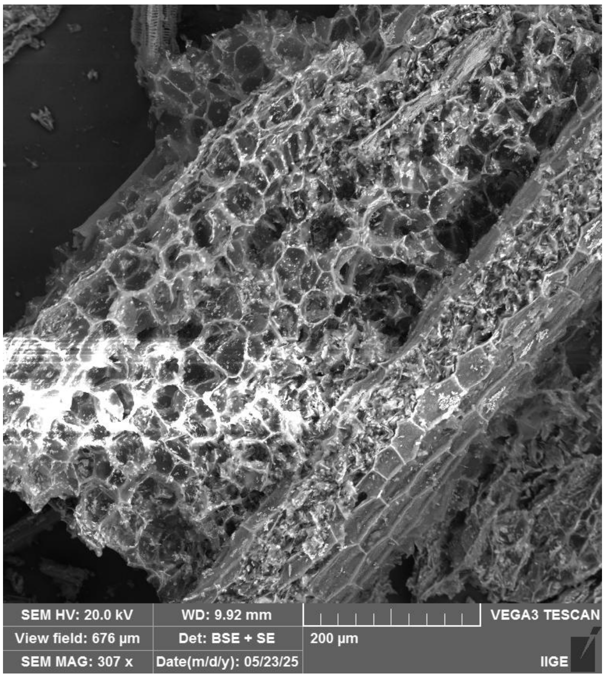

Figure S6. SEM of BC-OFMSW at 200 μm.

S2.9. Surface Area (BET) and Pore Size Distribution (PSD) of BC-OFMSW and BCA-OFMSW

The BET and PSD analysis BC-OFMSW data is available in the file "DATA_BET_BCA-OFMSW.csv".

The BET and PSD analysis BCA-OFMSW data is available in the file "DATA_BET_BC-OFMSW.csv".

S2.10. pH, Conductivity and Point of Zero Charge (pH_{pzc})

Table S15. Point of Zero Charge (pHpzc) content data.

Sample	Replicate	pH theorical	pH experimental	pHf-pHi	SD
BCA-OFMSW	1	2	2.97	0.9600	0.0141
	2	2	2.95		
	1	4	10.37	6.3800	0.0141
	2	4	10.39		
	1	6	10.34	4.2500	0.1273
	2	6	10.16		
	1	8	10.41	2.2550	0.2192
	2	8	10.1		
	1	10	10.41	0.3650	0.0636
	2	10	10.32		
	1	12	11.9	-0.1300	0.0424
	2	12	11.84		

Table S16. Conductivity content data.

Sample	Replicate	CE, uS cm^{-1}	Media	SD
BCA-OFMSW	1	527.00	536.00	16.46
	2	526.00		
	3	555.00		

Table S17. pH content data.

Sample	Replicate	pH	Media	SD
	1	9.82		
BCA-OFMSW	2	9.86	9.86	0.04
	3	9.90		

S2.11. Effect of Operational Parameters on the Adsorption Process

S2.11.1. Effect of pH

Table S18. Effect of pH content data.

pH	$[C_e]$ mg L^{-1}	Media	SD	Yield %	Media	SD	q_e mg g^{-1}	Media	SD
2	140.0205	142.6158	2.5953	29.9383	28.6398	1.2986	57.4211	55.8148	1.6062
2	145.2110			27.3412			54.2086		
3	148.7853	149.8497	1.0645	25.5527	25.0201	0.5326	51.7931	50.1719	1.6212
3	150.9142			24.4875			48.5506		
4	175.5420	176.0938	0.5518	12.1645	11.8884	0.2761	23.5574	23.0665	0.4909
4	176.6456			11.6123			22.5755		
5	189.5988	191.0890	1.4902	5.1310	4.3853	0.7456	9.9172	8.5313	1.3859
5	192.5792			3.6397			7.1454		
6	196.6186	195.3597	1.2589	1.6185	2.2484	0.6299	3.1588	4.3937	1.2349
6	194.1009			2.8783			5.6286		

S2.11.2. Effect of Contact Time

Table S19. Effect of contact time content data.

Time min	$[C_e]$ mg L^{-1}	Media	SD	Yield %	Media	SD	q_e mg g^{-1}	Media	SD
10	158.5258	157.5103	1.0155	20.6789	21.1870	0.5081	41.4934	41.1220	0.3715
10	156.4947			21.6952			40.7505		
60	159.8798	155.3664	4.5135	20.0014	22.2598	2.2584	40.6234	45.0594	4.4360
60	150.8529			24.5182			49.4953		
120	138.2151	137.1996	1.0155	30.8417	31.3498	0.5081	58.1492	61.2958	3.1467
120	136.1840			31.8580			64.4425		
180	146.0009	137.4253	8.5756	26.9460	31.2369	4.2910	50.9966	60.2359	9.2393
180	128.8496			35.5279			69.4752		
240	135.5070	131.8398	3.6672	32.1967	34.0317	1.8349	62.3510	65.5712	3.2202
240	128.1726			35.8666			68.7914		

S2.11.3. Effect of Adsorbent Dosage

Table S20. Effect of adsorbent dosage content data.

Dosage mg	[C_e] mg L^{-1}	Media	SD	Yield %	Media	SD	q_e mg g^{-1}	Media	SD
11.0000	185.1554	186.8479	1.6926	7.3543	6.5074	0.8469	66.8086	61.6862	5.1224
10.0000	188.5405			5.6605			56.5638		
20.5000	178.7236	176.2976	2.4260	10.5726	11.7864	1.2139	51.5356	57.6080	6.0724
20.4000	173.8717			13.0003			63.6804		
50.2000	132.3476	145.4367	13.0891	33.7776	27.2283	6.5494	67.2367	53.4873	13.7494
52.0000	158.5258			20.6789			39.7379		
103.9000	122.4179	113.6166	8.8013	38.7461	43.1500	4.4039	37.2644	42.1331	4.8688
101.1000	104.8153			47.5539			47.0019		
199.1000	36.4360	36.1539	0.2821	81.7686	81.9098	0.1411	41.0390	40.7641	0.2749
202.5000	35.8718			82.0509			40.4892		

S2.11.4. Effect of Initial Concentration Cr(VI)

Table S21. Effect of initial concentration Cr(VI) content data.

[Co] mg L^{-1}	[Ce] mg L^{-1}	Media	SD	Yield %	Media	SD	q_e mg g^{-1}	Media	SD
300	150.4357	159.5020	9.0663	49.8548	46.8327	3.0221	74.6329	69.5934	5.0395
300	168.5683			43.8106			64.5539		
250	122.7564	130.6191	7.8627	50.8974	47.7523	3.1451	60.2479	58.2000	2.0479
250	138.4819			44.6073			56.1521		
200	92.6126	91.4842	1.1284	53.6937	54.2579	0.5642	51.4308	52.4318	1.0010
200	90.3558			54.8221			53.4328		
150	53.0991	55.9313	2.8322	64.6006	62.7125	1.8881	47.0850	46.5124	0.5726
150	58.7635			60.8243			45.9398		
100	18.1064	19.7945	1.6880	81.8936	80.2055	1.6880	39.4478	38.6529	0.7949
100	21.4825			78.5175			37.8580		

S2.12. Adsorption Isotherms

Table S22. Adsorption isotherms content data.

**Langmuir**	
Equation	(qm*K*Ce)/(1+K*Ce)
Plot	q, mg g-1
qm	56.86444 ± 3.57428
K	0.09815 ± 0.03267
R-Square (COD)	0.8453
Adj. R-Square	0.79374
**Freundlich**	
Equation	KF*((Ce)^(1/n))
Plot	q, mg g-1
KF	20.15826 ± 2.10219
n	4.73098 ± 0.58139
R-Square (COD)	0.95908
Adj. R-Square	0.94544

S2.13. Adsorption Kinetics

Table S23. Adsorption kinetics content data.

**[C_e] mg L^{-1}**	**Media**	**SD**	**Yield %**	**Media**	**SD**	**q_e mg g^{-1}**	**Media**	**SD**
35.1674	35.6820	0.5145	64.9430	64.4301	0.5129	31.3511	30.6705	0.6806
36.1965			63.9172			29.9900		
29.8415	29.7603	0.0812	70.2522	70.3332	0.0810	35.9926	34.6087	1.3839
29.6790			70.4142			33.2248		
16.7163	17.6370	0.9208	83.3362	82.4183	0.9179	40.8996	40.6268	0.2728
18.5578			81.5005			40.3540		
8.3393	8.3122	0.0271	91.6869	91.7139	0.0270	45.9419	45.9325	0.0094
8.2851			91.7409			45.9231		
3.6452	4.3042	0.6590	96.3662	95.7093	0.6569	47.1100	45.5458	1.5643
4.9632			95.0524			43.9815		
2.0312	2.6125	0.5813	97.9752	97.3957	0.5795	49.4883	47.8462	1.6421
3.1939			96.8161			46.2042		

Table S24. Adsorption kinetics models content data.

**Pseudo Second Order**	
Equation	((qe^2)*(K2)*t)/(1+(qe*K2*t))
Plot	q, mg g-1
qe	49.18584 ± 0.99043
K2	0.00478 ± 0.00155
R-Square (COD)	0.86739
Adj. R-Square	0.83423
**Difusion Intraparticle**	
Equation	Ki*Sqrt(t)+Ci
Plot	q, mg g-1
Ki	2.32976 ± 0.37261
Ci	27.88507 ± 2.88518
R-Square (COD)	0.90718
Adj. R-Square	0.88398
**Elovich**	
Equation	(1/B)*(ln(A*B*t+1))
Plot	q, mg g-1
A	119.7756 ± 69.72037
B	0.15239 ± 0.0148
R-Square (COD)	0.96538
Adj. R-Square	0.95673

The adsorption kinetics of Cr(VI) onto BCA-OFMSW were evaluated over a contact-time interval of 0–180 min.

S2.14. Adsorption Thermodynamics

Table S25. Adsorption thermodynamics content data.

T<br>K	[C$_o$]<br>mg L^{-1}	[C$_e$]<br>mg L^{-1}	Media	SD	Yield<br>%	Media	SD	q$_e$<br>mg g^{-1}	Media	SD
**298.15**	25	0.4858	0.5670	0.0812	98.0568	97.7318	0.3250	11.2039	11.1064	0.0975
	25	0.6483			97.4069			11.0089		
	50	6.0645	6.0284	0.0361	87.8711	87.9433	0.0722	21.7934	21.8222	0.0287
	50	5.9922			88.0155			21.8509		
	100	37.3339	38.2546	0.9208	62.6661	61.7454	0.9208	29.5316	28.9507	0.5810
	100	39.1754			60.8246			28.3697		
	150	71.6700	71.8957	0.2257	52.2200	52.0696	0.1504	37.7312	37.8604	0.1292
	150	72.1213			51.9191			37.9896		
	200	119.3201	109.2776	10.0425	40.3400	45.3612	5.0213	37.4211	43.4807	6.0596
	200	99.2351			50.3825			49.5403		
	300	195.6964	187.1659	8.5305	34.7679	37.6114	2.8435	48.1994	52.2716	4.0722
	300	178.6355			40.4548			56.3438		
**308.15**	25	-0.2364	-0.2364	0.0000	100.9455	100.9455	0.0000	11.7160	12.0861	0.3701
	25	-0.2364			100.9455			12.4563		
	50	2.1468	2.1829	0.0361	95.7065	95.6343	0.0722	21.9914	21.8847	0.1067
	50	2.2190			95.5621			21.7780		
	100	12.2750	11.8327	0.4423	87.7250	88.1673	0.4423	40.0937	41.3063	1.2127
	100	11.3904			88.6096			42.5190		
	150	44.7360	44.7811	0.0451	70.1760	70.1459	0.0301	49.3271	49.4687	0.1416
	150	44.8263			70.1158			49.6102		
	200	75.4264	75.5957	0.1693	62.2868	62.2022	0.0846	60.8269	59.2785	1.5484
	200	75.7649			62.1175			57.7300		
	300	160.0399	160.3107	0.2708	46.6534	46.5631	0.0903	68.0078	65.7476	2.2602
	300	160.5815			46.4728			63.4875		
**318.15**	25	-0.2364	-0.2273	0.0090	100.9455	100.9093	0.0361	12.5554	12.4587	0.0967
	25	-0.2183			100.8732			12.3619		
	50	1.7135	1.9752	0.2618	96.5731	96.0495	0.5236	22.5638	22.5894	0.0256
	50	2.2370			95.5259			22.6150		
	100	15.6692	14.1887	1.4804	84.3308	85.8113	1.4804	40.1958	44.0003	3.8045
	100	12.7083			87.2917			47.8049		
	150	24.8586	26.3571	1.4985	83.4276	82.4286	0.9990	61.9512	61.4507	0.5004
	150	27.8556			81.4296			60.9503		
	200	33.7773	49.9861	16.2089	83.1114	75.0069	8.1044	82.7802	74.3795	8.4007
	200	66.1950			66.9025			65.9788		
	300	114.2731	116.4396	2.1665	61.9090	61.1868	0.7222	89.3777	89.1481	0.2296
	300	118.6061			60.4646			88.9186		

Table S26. Calibration curve used for the quantification of Cr(VI) in adsorption tests.

Concentration Cr(VI) mg L^{-1}	Replicate	Abs λ=350nm	Media	SD
	1	0.5860		
25.0000	2	0.5900	0.5890	0.0022
	3	0.5910		
	1	0.5170		
20.0053	2	0.5120	0.5143	0.0021
	3	0.5140		
	1	0.4440		
14.9890	2	0.4450	0.4447	0.0005
	3	0.4450		
	1	0.3620		
11.9912	2	0.3680	0.3640	0.0028
	3	0.3620		
	1	0.2920		
8.9934	2	0.2910	0.2913	0.0005
	3	0.2910		
	1	0.1800		
5.9956	2	0.1790	0.1790	0.0008
	3	0.1780		
	1	0.0930		
2.9978	2	0.0920	0.0923	0.0005
	3	0.0920		
	1	1.0080		
34.9943	2	1.0090	1.0083	0.0005
	3	1.0080		
	1	1.4250		
50.0033	2	1.4200	1.4217	0.0024
	3	1.4200		

S2.15. Post-Adsorption Structural Characterization of BCA-Cr

The XRD analysis of BCA-Cr data is available in the file "DATA_DRX_BCA-Cr.csv"

The SEM-EDS analysis of BCA-OFMSW data is available in the file "DATA_SEM-EDS_BCA-OFMSW.csv"

The SEM-EDS analysis of BCA-Cr data is available in the file "DATA_SEM-EDS_BCA-Cr.csv"

Table S27. Elemental composition (wt%) of BCA-OFMSW before and after Cr(VI) adsorption, determined by EDS analysis.

Element	Spot 1	Spot 2	Spot 3	Spot 4	Spot 5	Spot 6	Spot 7	Spot 8	Media	sd
	**BCA-OFMSW**									
	**%wt**									
C	59.59	56.47	75.71	80.11	70.47	-	62.86	54.97	57.52	24.95
O	29.75	24.35	19.55	12.87	24.62	57.46	21.61	21.94	26.52	13.40
Ca	5.52	-	1.16	2.27	2.42	13.81	6.48	11.87	5.44	5.06
Mg	2.14	-	1.37	1.74	1.27	6.06	1.64	-	1.78	1.90
Si	1.52	-	0.68	1.00	-	5.16	2.18	2.04	1.57	1.67
N	-	19.19	-	-	-	10.32	-	-	3.69	7.23
P	0.73	-	-	0.58	1.22	5.43	0.92	3.12	1.50	1.87
Cl	-	-	-	-	-	1.09	1.53	2.53	0.64	0.97
Al	-	-	-	-	-	0.67	-	-	0.08	0.24
K	0.75	-	0.86	1.44	-	-	2.77	3.53	1.17	1.34
S	-	-	0.68	-	-	-	-	-	0.09	0.24
	**BCA-Cr**									
C	59.17	56.97	53.44	50.46	55.71	39.52	53.47	-	52.68	6.44
O	26.25	19.13	22.97	20.98	24.31	32.97	22.05	-	24.09	4.53
Cr	7.29	18.72	15.86	21.47	15.15	8.15	18.85	-	15.07	5.44
N	-	-	-	-	-	9.37	-	-	1.34	3.54
Si	1.75	2.37	2.94	2.56	2.54	4.79	2.17	-	2.73	0.98
P	1.27	2.81	2.41	2.69	2.30	1.27	2.23	-	2.14	0.63
Mg	0.95	-	-	-	-	0.83	-	-	0.25	0.44
Ca	0.90	-	1.44	1.83	-	1.78	1.22	-	1.02	0.77
Al	0.43	-	0.93	-	-	1.32	-	-	0.38	0.54
S	-	-	-	-	-	-	-	-	0.29	0.76

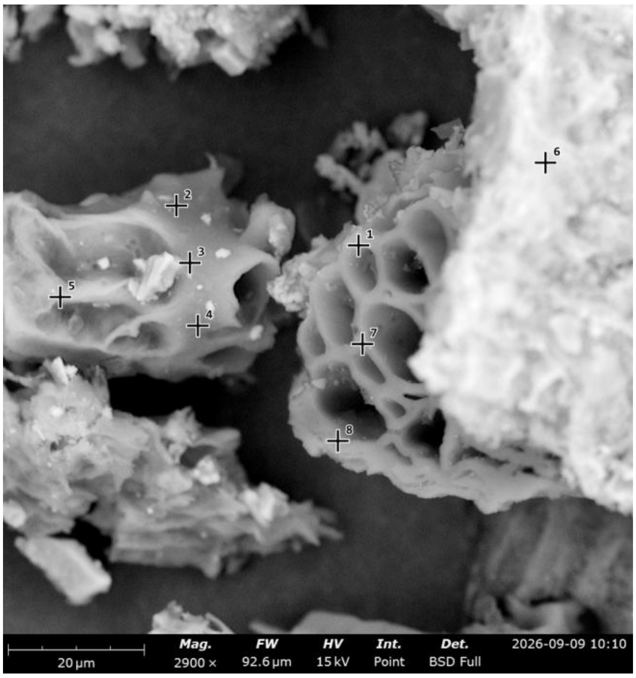

Figure S7. SEM-EDS of BCA-OFMSW at 20 μm.

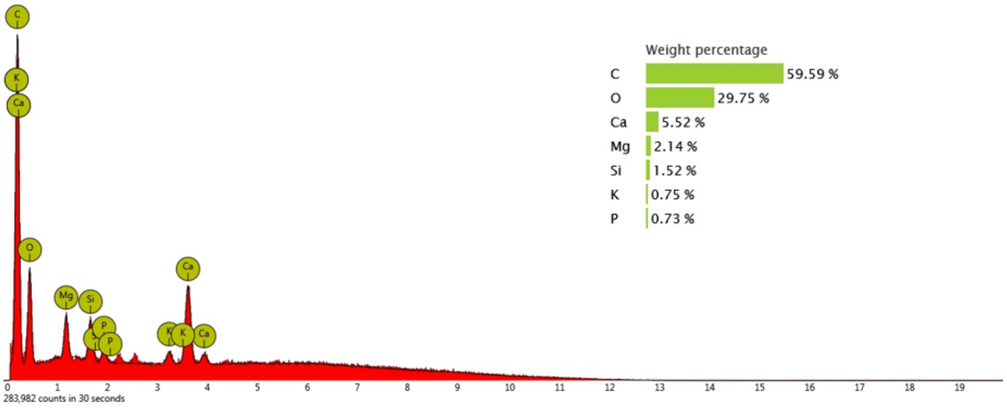

Figure S8. Spectrum of BCA-OFMSW, spot1.

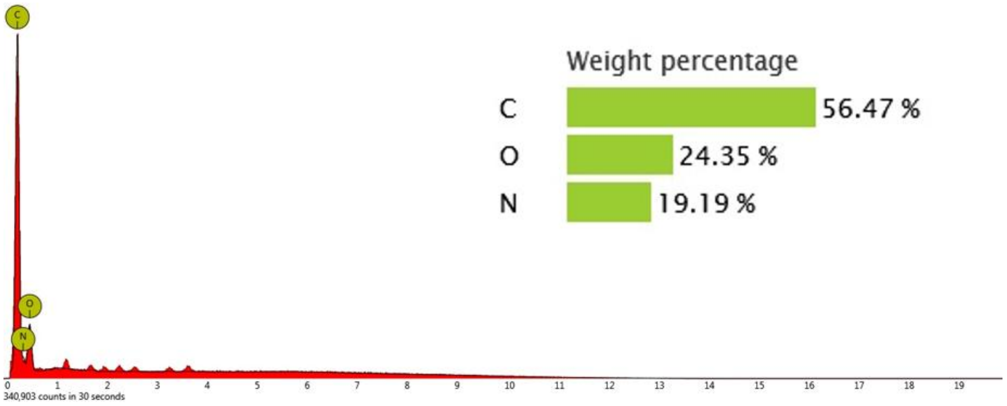

Figure S9. Spectrum of BCA-OFMSW, spot2.

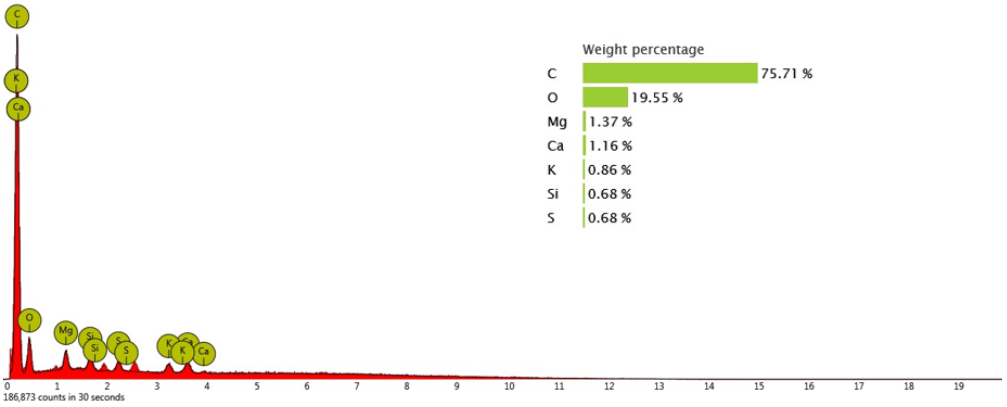

Figure S10. Spectrum of BCA-OFMSW, spot3.

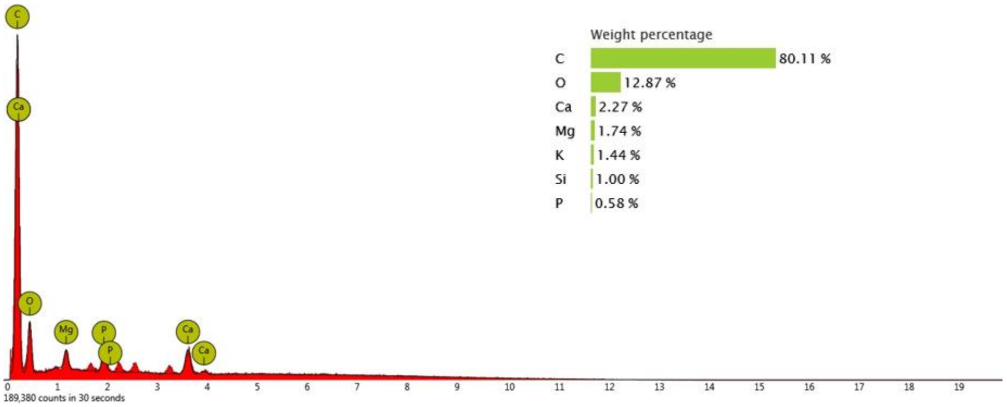

Figure S11. Spectrum of BCA-OFMSW, spot4.

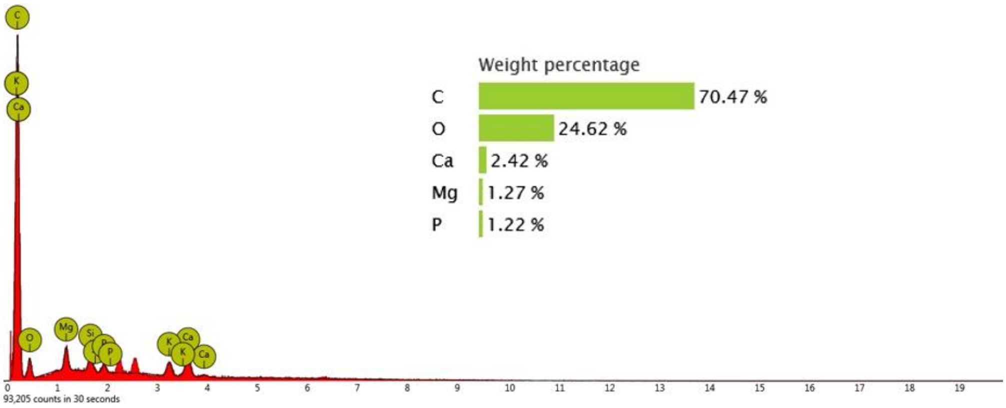

Figure S12. Spectrum of BCA-OFMSW, spot5.

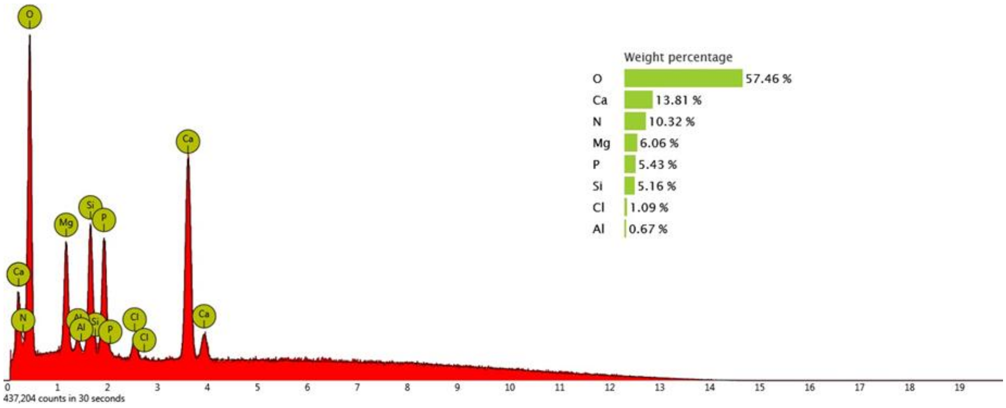

Figure S13. Spectrum of BCA-OFMSW, spot6.

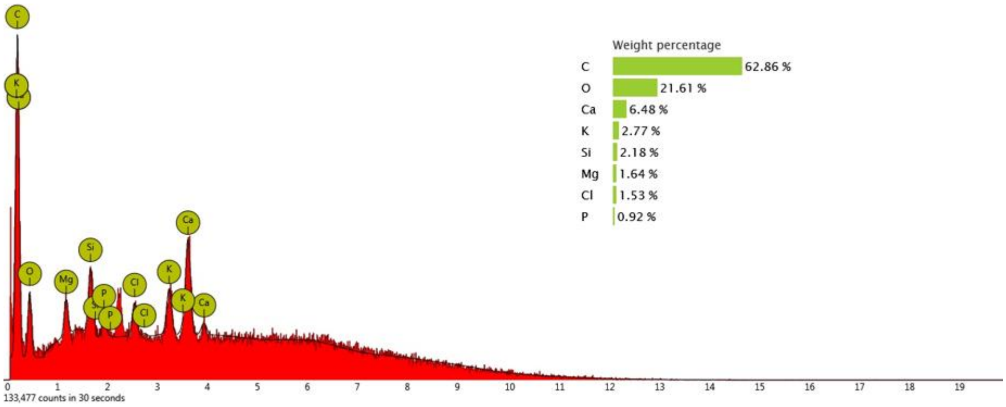

Figure S14. Spectrum of BCA-OFMSW, spot7.

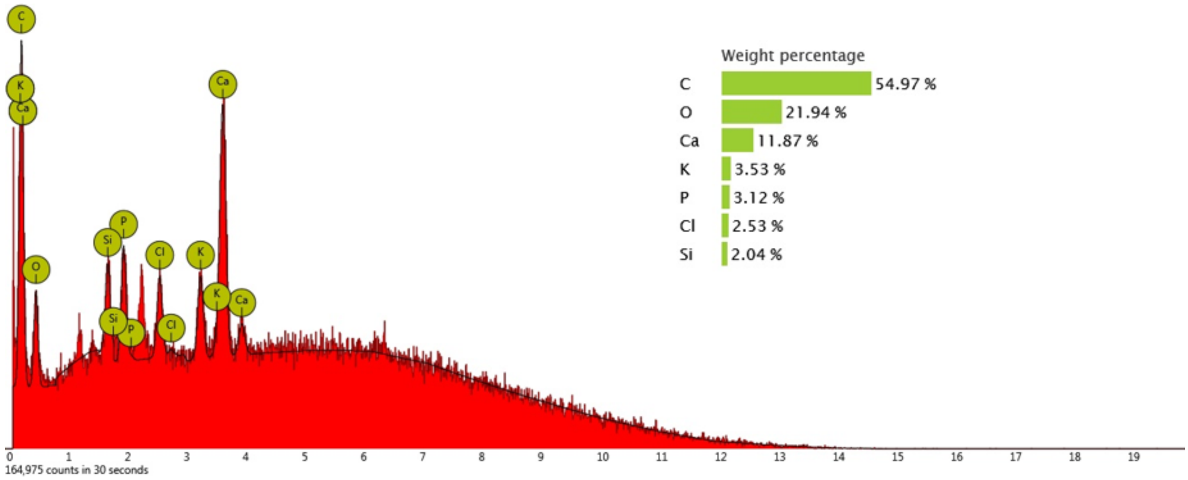

Figure S15. Spectrum of BCA-OFMSW, spot8.

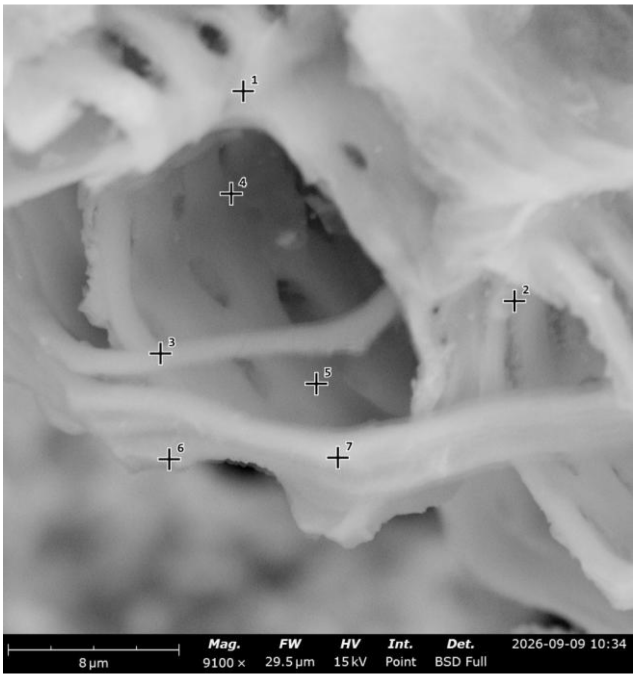

Figure S16. SEM-EDS of BCA-Cr at 20 μm.

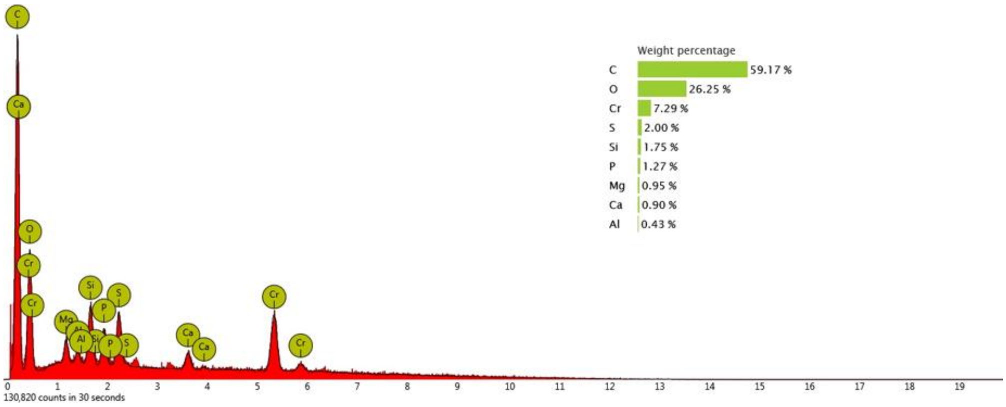

Figure S17. Spectrum of BCA-Cr, spot1.

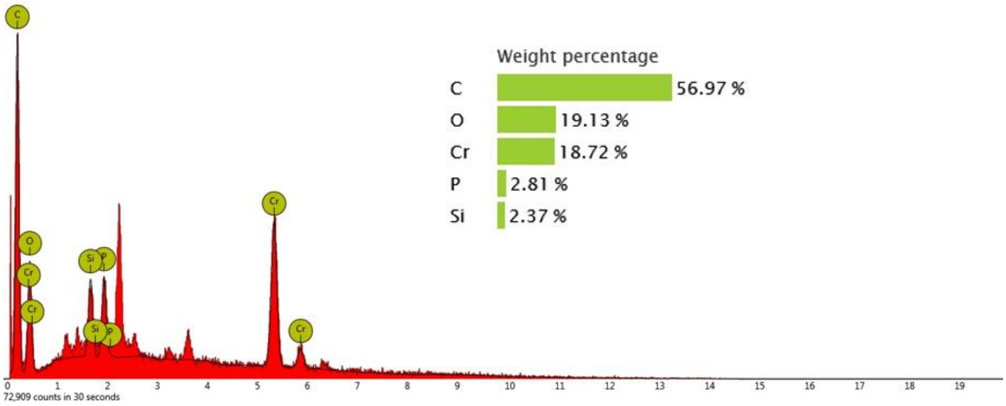

Figure S18. Spectrum of BCA-Cr, spot2.

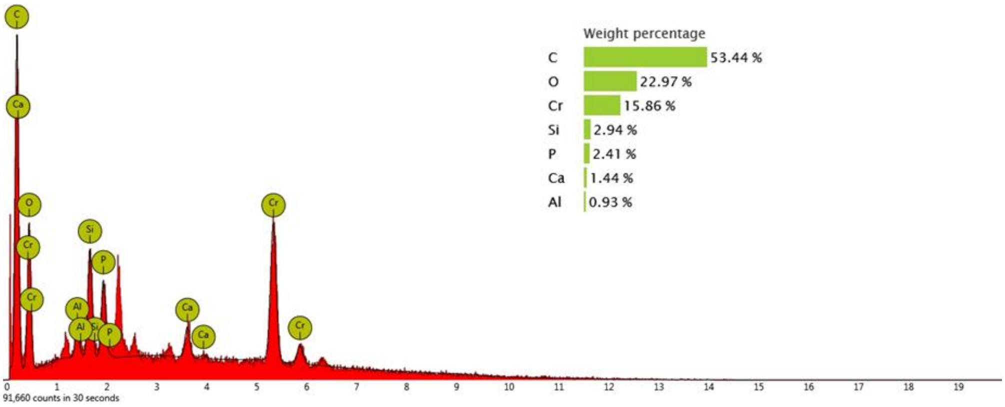

Figure S19. Spectrum of BCA-Cr, spot3.

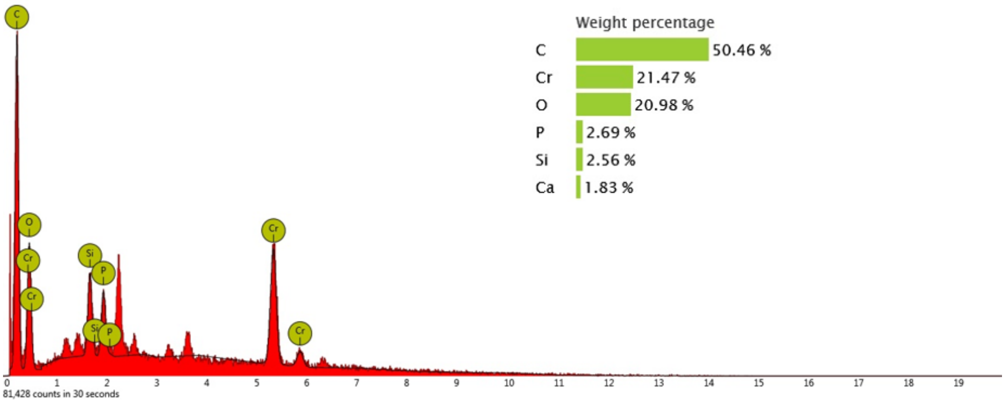

Figure S20. Spectrum of BCA-Cr, spot4.

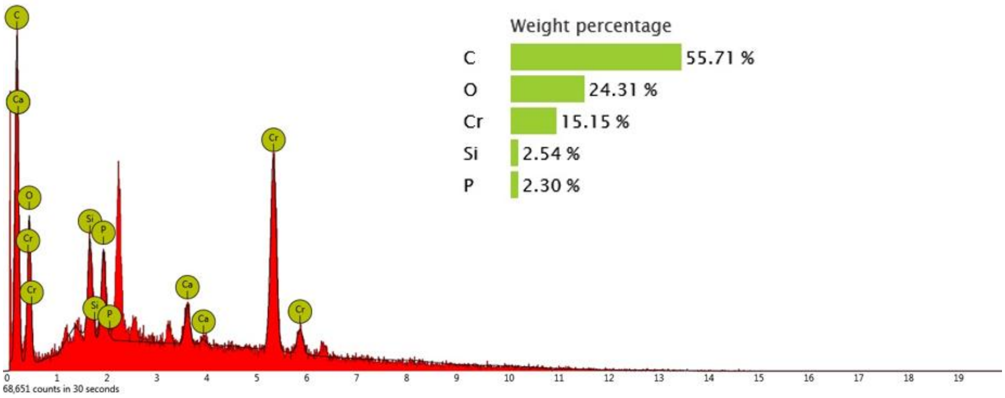

Figure S21. Spectrum of BCA-Cr, spot5.

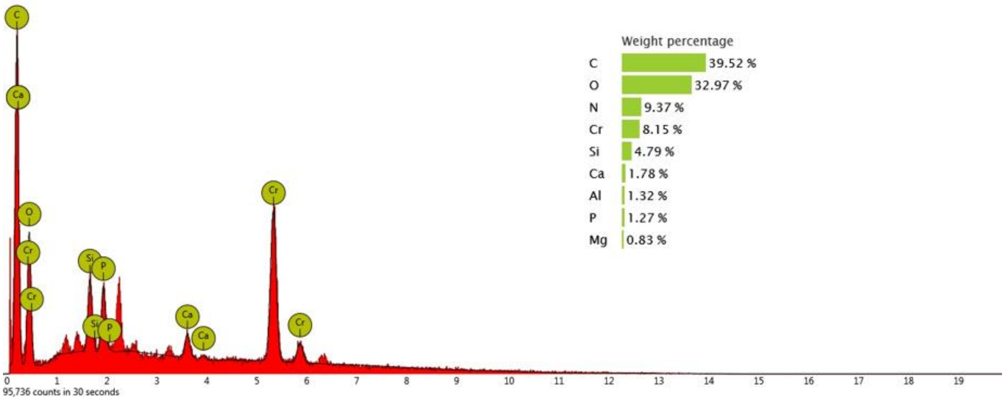

Figure S22. Spectrum of BCA-Cr, spot6.

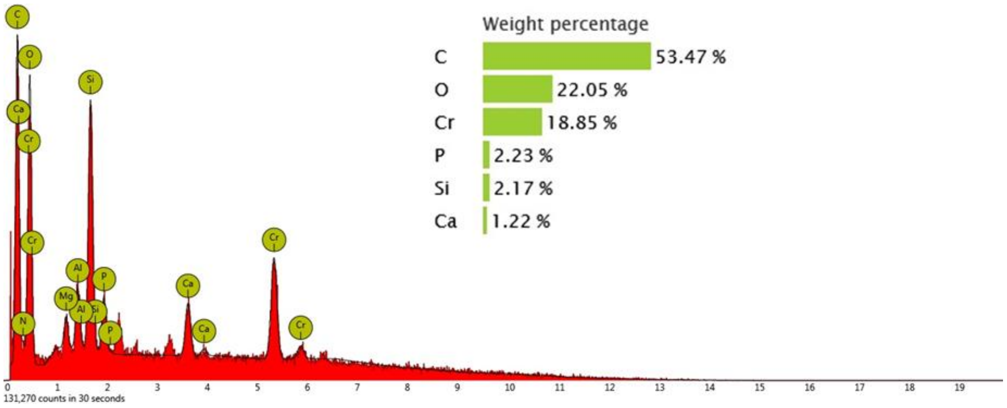

Figure S23. Spectrum of BCA-Cr, spot7.

S3. Methods

All methods applied to collect and treat the data are presented and explained in the Materials and Methods section of the associated manuscript. This data descriptor does not introduce any new methods.

References

1. Reif, B.M.; Murray, B.P. Chromium Toxicity. StatPearls 2024. Available online https://pubmed.ncbi.nlm.nih.gov/38261677/ (accessed on 24 August 2026).

Disclaimer/Publisher's Note: The statements, opinions and data contained in all publications are solely those of the individual author(s) and contributor(s) and not of MDPI and/or the editor(s). MDPI and/or the editor(s) disclaim responsibility for any injury to people or property resulting from any ideas, methods, instructions or products referred to in the content.
